# PeptideMTR: Scaling SMILES-Based Language Models for Therapeutic Peptide Engineering

**DOI:** 10.64898/2026.01.06.697994

**Published:** 2026-01-07

**Authors:** Aaron L. Feller, Maxim Secor, Sebastian Swanson, Claus O. Wilke, Kristine Deibler

**Affiliations:** 1 Molecular AI, Novo Nordisk, 33 Hayden Ave, Lexington, 02421, MA, USA.; 2 Integrative Biology, The University of Texas at Austin, 2500 Speedway, Austin, 78712, TX, USA.

**Keywords:** chemical language models, peptide representation learning, multi-task regression, foundation models, scaling laws, machine learning for drug discovery, noncanonical peptides

## Abstract

Therapeutic peptides occupy a unique region of chemical space, combining the modularity of proteins with the versatility of small molecules. However, existing foundation models struggle to represent this domain: protein language models are confined to canonical residues, while small-molecule models often lack the contextual range required for peptide sequences. Here we introduce PeptideMTR, a suite of nine SMILES-based chemical language models (32M–337M parameters) pretrained on peptide and small molecule data with three objectives: masked language modeling, multi-task regression to physicochemical descriptors, and a combined objective. Systematic evaluation on membrane permeability prediction reveals a distinct scaling transition: at smaller scales, descriptor-guided pretraining provides a crucial inductive bias, grounding embeddings in physicochemical space; however, as capacity increases, purely self-supervised models recover equivalent predictive capability, suggesting that large models spontaneously internalize these physicochemical priors. PeptideMTR outperforms molecular fingerprints and specialized architectures on diverse peptide tasks including aggregation propensity, tumor homing, cell penetration, and antimicrobial activity. We release PeptideMTR as an open, scalable resource for therapeutic peptide engineering.

## 1 Introduction

Peptides are an increasingly important therapeutic class [1–6], occupying a chemical space between small molecules and proteins. They combine the modularity of biological polymers with the synthetic accessibility of small molecules, offering immense therapeutic potential. Drug-like peptides frequently incorporate noncanonical residues [7], cyclization [8, 9], and diverse chemical modifications [10, 11] that extend their utility beyond natural, linear sequences. Recent proteogenomic studies have revealed that such non-canonical peptides arise frequently in human biology via RNA circularization and frameshifts [12]. However, these features challenge existing representation-learning paradigms: protein language models (PLMs) are restricted to fixed amino-acid alphabets and cannot encode noncanonical or chemically modified residues [13], and chemical language models (CLMs) are typically trained on small molecules and thus lack the context to interpret peptide-specific motifs and long-range dependencies [14, 15].

While foundation models have revolutionized protein and small-molecule engineering, the development of peptide specific models lags far behind these advances. Large-scale protein corpora such as UniProt [16] have enabled self-supervised PLMs that learn structural and functional constraints directly from sequence data. Architectures like the ESM family [17–19] and ProtTrans [20] demonstrate that transformers can capture deep dependencies predictive of structure [21–23], variant effects [24, 25], and molecular function [26, 27]. Simultaneously, CLMs based on symbolic representations such as SMILES or SELFIES [28, 29] have successfully encoded chemical syntax through masked or autoregressive objectives [30–32]. Notably, extensions such as ChemBERTa-2 [33] or ProLLaMA [34] suggest that augmenting self-supervision with regression to physicochemical or domain descriptors can introduce a valuable inductive bias, improving generalization. Despite these advances, deep learning has shown only minimal improvement over molecular fingerprints for predicting peptide properties [35, 36].

Here, we introduce PeptideMTR, a suite of nine SMILES-based transformer encoders [37] designed to advance therapeutic peptide modeling. Building on our previous work that resulted in PeptideCLM [38], we train models across a parameter range of 32 to 337 million using three distinct objectives: masked language modeling (MLM), multi-task regression (MTR) [39] to RDKit-derived descriptors [40], and a dual objective, combining both. This systematic approach allows us to decouple the effects of model scale and inductive bias on representation learning. Our evaluation on peptide-property prediction reveals a critical scaling transition: while descriptor-guided pretraining (MTR) significantly benefits smaller models, larger architectures trained solely on MLM spontaneously recover these physicochemical relationships, competing with explicit supervision on downstream evaluations. We find that these models are able to outperform molecular fingerprints, highlighting that increasing model capacity can improve predictive performance. Together, these results position PeptideMTR as both a valuable open resource for peptide engineering and a framework for understanding how capacity and objective functions interact in peptide chemical space.

## 2 Results

### 2.1 Model architecture and training design

The PeptideMTR suite is a modular collection of chemical language models (CLMs) designed to represent, analyze, and fine-tune on drug-like peptides. It provides a multi-scale framework for learning from both symbolic and physicochemical representations, combining scalable transformer architectures with interpretable tokenization strategies.

To explore the impact of parameter scale and pretraining objective on predictive capability, we trained nine models systematically varying in size—32M, 114M, and 337M parameters—and in pretraining objective: masked-language modeling (MLM), multi-task regression (MTR) to physicochemical descriptors, or a combined dual objective. This controlled design provides insight into model capacity and learning paradigm.

**Fig. 1:**
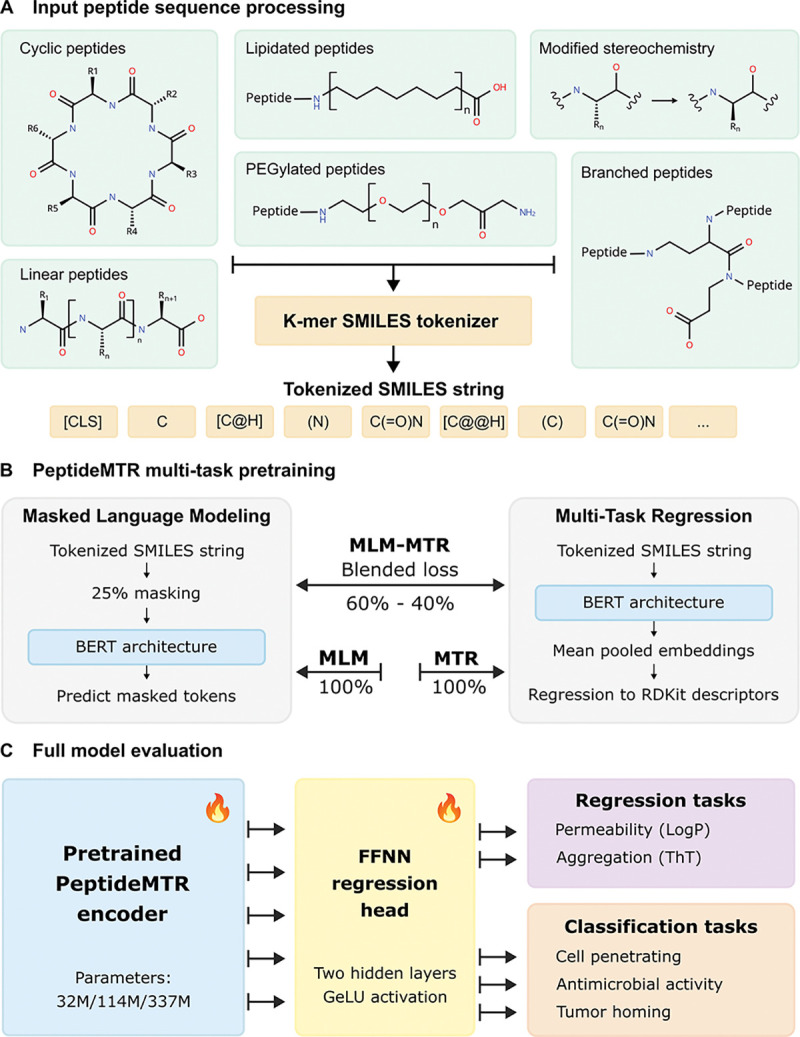
Overview of the PeptideMTR Chemical Language Model Framework. (A) Input processing encodes diverse non-canonical peptide structures into compact tokenizations via a k-mer SMILES Tokenizer. (B) Pretraining objectives include Masked Language Modeling (MLM), Multi-Task Regression (MTR) to 99 RDKit physicochemical descriptors, and a blended loss (MLM-MTR). (C) Evaluation workflow showing the fine-tuning of the pretrained PeptideMTR encoder on downstream regression and classification tasks using a standardized feed-forward head.

The models are capable of encoding canonical and noncanonical amino acids, cyclic scaffolds, and diverse backbone or side-chain modifications (including lipidation and PEGylation) directly from SMILES strings (Fig. 1A). Each transformer layer incorporates multi-head self-attention with rotary positional embeddings, SwiGLU feed-forward blocks [41], pre-layer normalization [42], residual skip connections, and dropout. Model depth and hidden dimension scale with parameter count, maintaining a per-head dimensionality of 64. Pretraining followed one of three objectives (Fig. 1B): in MLM, random or span masking [43] encouraged contextual reconstruction; in MTR, models were trained to regress to 99 RDKit-derived physicochemical descriptors (Tab. S1) from a mean-pooled embedding (Fig. S1); and in MLM–MTR, both losses were calculated from masked inputs and optimized jointly. The most robust predictions came from finetuning the full model, with a 2-hidden layer feed-forward regression head employing GeLU activation (Fig. 1C).

### 2.2 Pretraining data and tokenization schema

**Fig. 2:**
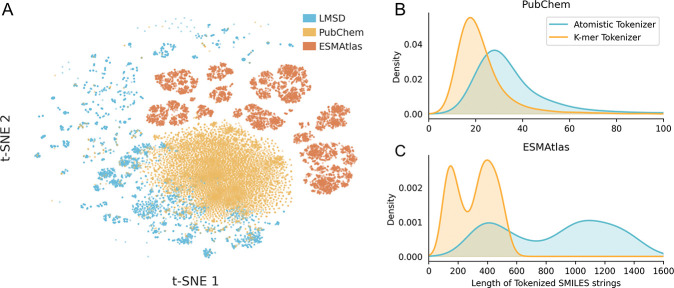
Overview of pretraining data, tokenization compression, and model performance. (A) A 2D t-SNE projection of Morgan Fingerprints from the pretraining data built from three datasets (LMSD, PubChem, and ESMAtlas) highlighting distinct chemical subspaces. Length distribution of tokenized molecules for (B) small molecules and (C) peptides under atomistic and k-mer tokenization schemes. K-mer tokenization substantially compresses SMILES length while preserving distribution shape.

We pretrained nine models using a BERT-style transformer encoder with a composite dataset consisting of lipids from the LIPID MAPS Structure Database (LMSD; *n* = 50, 450) [44], small molecules from PubChem (*n* = 108, 583, 157) [45], and peptides from ESMAtlas (*n* = 9, 634, 945) [18]. These datasets inhabit unique regions of chemical space, as can be seen from a 2D t-SNE visualization (Fig. 2A) of Morgan Fingerprints [46] of each molecule.

When encoding peptides, care must be taken to reduce token count to reduce computational complexity of the model. At the same time, it is important to ensure chemical compatibility of tokens with SMILES syntax. Thus, we created a hand-tuned tokenizer built on a custom k-mer SMILES vocabulary with defined filtering rules (Tab. S2 & S3). This tokenizer forms the interface between symbolic and numeric representations, mapping discrete chemical syntax into a continuous embedding manifold.

Relative to the standard DeepChem atom-level tokenizer [47], our k-mer strategy reduced sequence lengths by 38% for small molecules and 64% for natural peptides (Fig. 2B). Crucially, this compression came at no cost to accuracy; benchmarking models trained with either tokenizer revealed equivalent performance on membrane permeability prediction (Table S8). Thus, the k-mer approach significantly lowered the computational overhead of self-attention while preserving the chemical integrity of the representation.

To isolate the effects of scale and pretraining objective, each model was trained and evaluated under identical data composition and hyperparameters (Tab. S4). This allowed a direct examination of how model capacity and property-guided regression influence transfer learning.

Fine-tuning workflows and hyperparameters (Tab. S5) were standardized through nested cross-validation and ensemble averaging, ensuring robust estimates of performance on peptide-specific endpoints such as membrane permeability and aggregation. The performance of PeptideMTR models was evaluated across multiple downstream peptide-property prediction tasks to assess the effects of model scale, pretraining objective, and embedding transferability.

### 2.3 Effect of pretraining scheme and model scale on predicting passive diffusion of cyclic peptides

We evaluated PeptideMTR using cyclic-peptide permeability data from CycPeptMPDB [48], a dataset with robust splits for benchmarking [38]. Model selection was informed by ablation studies that compared tokenization, masking, and pretraining schemes across three model sizes (Tab. S6). This resulted in selecting the nine models that we rigorously evaluate throughout this work.

**Fig. 3:**
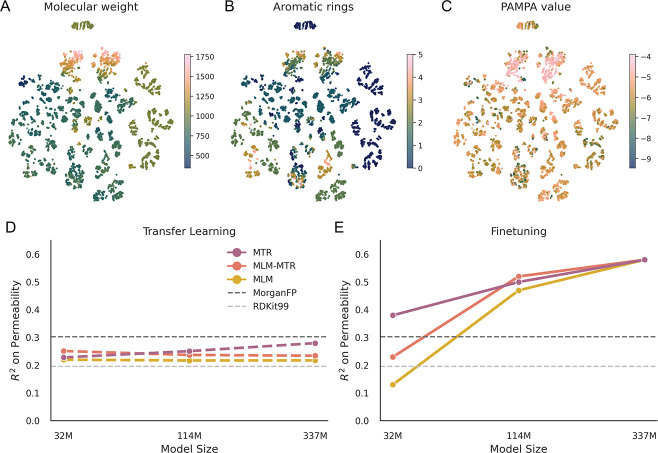
Analysis of pretrained model embeddings and predictive capability on cyclic peptide PAMPA data. A single t-SNE projection of 337M MLM-MTR embeddings, colored by (A) molecular weight, (B) aromatic ring count, and (C) measured PAMPA permeability, showing structural clustering. Line plots show a comparison of Transfer Learning (frozen embeddings) vs. Fine-tuning where (D) linear probes on frozen features yield static performance, while (E) full fine-tuning reveals a scaling transition where large self-supervised models recover the performance of descriptor-guided models.

To visualize the learned chemical space, we projected embeddings from the 337M MLM-MTR model, and saw that the model was able to organize the chemical manifold by physicochemical properties on which it was trained, such as molecular weight and aromaticity (Fig. 3A, B). This same manifold organized into clusters that separated by measured PAMPA permeability (Fig. 3C). Examination of additional descriptors confirmed that the model simultaneously encodes multiple physicochemical dimensions, with distinct clustering patterns emerging for each property within the same embedding space (Fig. S2).

To disentangle the effects of representational quality versus model capacity, we compared layer-wise transfer learning (linear probing on frozen features) against full-model fine-tuning. Transfer learning yielded consistently lower performance (*R*^2^ < 0.3) regardless of scale, suggesting that permeability relies on complex, non-linear features not easily accessed by a linear probe (Fig. 3D).

While the final hidden layer is typically used for feature extraction, it is not always the optimal source of information for specific physicochemical properties. To investigate the impact of layer choice, we trained linear probes on every layer of the model to predict membrane permeability. We observed that layer selection significantly impacted performance in smaller models, where the final layer often underperformed compared to intermediate representations (Fig. S3). In contrast, larger models exhibited high stability across depths, with negligible performance gaps between the final layer and the optimal intermediate layer.

However, fine-tuning revealed a critical scaling transition. At the smallest model size (32M), inductive bias is dominant: models pretrained with explicit physicochemical regression (MTR) significantly outperformed masked language modeling (MLM), achieving an *R*^2^ of ≈ 0.38 versus 0.13 (Fig. 3E). This indicates that when capacity is limited, explicit descriptor supervision is essential for grounding the representation.

As capacity increased to 337M parameters, this gap vanished. The MLM and MLM-MTR models converged with the MTR baseline, with all objectives reaching an *R*^2^ ≈ 0.58—nearly double that of molecular fingerprints (*R*^2^ ≈ 0.3). This convergence suggests that sufficiently large self-supervised models spontaneously internalize the physicochemical priors that must be explicitly taught to smaller models. While the increased dimensionality of the larger model inherently aids linear separability (Cover’s theorem), the fact that self-supervised representations match the performance of explicitly supervised baselines suggests that the model has learned to encode physicochemical-relevant features, rather than relying solely on dimension expansion.

### 2.4 Generalization to functional classification benchmarks: bioactivity and localization

To assess general ability of the model beyond permeability prediction, we evaluated PeptideMTR on three diverse benchmarks covering tumor homing, cell penetration, and antimicrobial activity (Tab. S7). These tasks span varying degrees of physicochemical complexity, from recognizing subtle sequence motifs in canonical peptides to modeling the distinct chemistry of non-canonical amino acids. All results were evaluated using Matthew’s Correlation Coefficient (MCC) to represent a balanced result for classification tasks that considers false positives and false negatives.

**Fig. 4:**
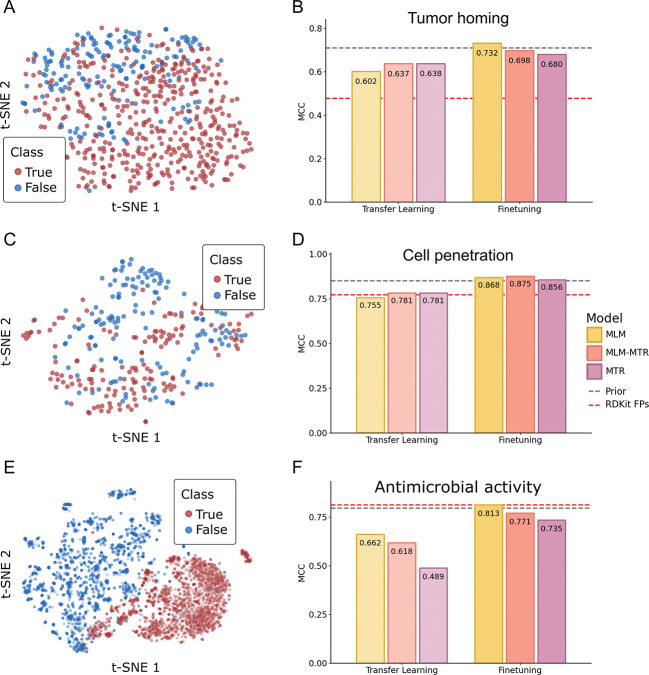
Evaluation of PeptideMTR on downstream peptide classification tasks. Left panels: t-SNE projections (A, C, E) of embeddings from the 337M MLM-MTR model, colored by class label (0 vs 1), showing distinct separation across all three bioactivity tasks. Bar plots of Matthews Correlation Coefficient (B, D, F) between Transfer Learning (linear probe) with random forest classifier and full fine-tuning with added regression head. Grey dashed lines indicate the performance of prior state-of-the-art methods. Red dashed lines indicate random forest using RDKit topological fingerprint (2048-bit, 2 bits/hash).

To compare the utility of static features against adaptive representations, we evaluated models via transfer learning (Random Forest model trained on static embeddings) and full-model fine-tuning (Fig. 4). For the fine-tuning tasks, we utilized a non-linear MLP head with two hidden layers, ReLU activations, and dropout, allowing the pretrained weights to be updated end-to-end.

Pretrained embeddings from PeptideMLM-MTR exhibit strong linear separability between positive and negative classes across all three classification benchmarks when projected using t-SNE (Fig. 4A, C, E). This latent structure translates into state-of-the-art fine-tuning performance that consistently outperforms specialized prior methods (Fig. 4B, D, F).

Tumor-Homing Peptides (THPs) are notoriously difficult to classify, due to the subtle recognition motifs (e.g., RGD, NGR) required to target tumor vasculature [49]. The prior state-of-the-art method, THPep, relied on hand-crafted feature engineering, specifically Pseudo Amino Acid Composition (PseAAC), coupled with Random Forest classifiers to achieve an MCC of ≈ 0.71 on independent tests [50]. Our MLM model learned these motif-driven features from SMILES alone, achieving an MCC of 0.732 without explicit feature extraction (Fig. 4B).

Cell-Penetrating Peptides (CPPs) were evaluated using the CellPPD-Mod dataset [51], which consists of peptides containing experimentally validated chemical modifications. Previous baselines required the extraction of 2D/3D chemical descriptors (e.g., PaDEL) because standard sequence models could not process the modified syntax. Our combined MLM-MTR objective achieved an MCC of 0.875, surpassing the descriptor-based Random Forest baselines (MCC ≈ 0.85) using only SMILES as inputs (Fig. 4D).

Antimicrobial Peptides (AMPs) provided the most rigorous test of chemical generalization. We utilized a benchmark created by He et al. [52], a dataset explicitly constructed to challenge models with peptides containing non-canonical amino acids. The model released with the benchmark dataset, AmpHGT, employs a complex Heterogeneous Graph Transformer to explicitly model atoms, fragments, and residues, and achieves an MCC of 0.797 in so-called “zero-shot” evaluations, where the model is finetuned to predict AMP activity even though it has not been exposed to non-canonical amino acids. Our model surpassed this specialized graph architecture with an MCC of 0.813 (Fig. 4F).

This result demonstrates that our SMILES-based encoding strategy captures the intrinsic chemistry of noncanonical amino acids using learned attention graphs rather than explicit molecular graphs.

### 2.5 Predicting peptide aggregation and formulation stability

A primary bottleneck in the development of peptide therapeutics is aggregation, which compromises efficacy, safety, and formulation-stability. Predicting this behavior is a critical challenge in pharmaceutical design, as it requires modeling many components from excipients to pH-dependent intermolecular interactions. To evaluate PeptideMTR in this regime, we fine-tuned the MLM-MTR models on a large in-house proprietary ThT fluorescence dataset. The ThT assay is a standard industrial benchmark for assessing lipid–peptide fibrillation and aggregation propensity [53]. This proprietary dataset comprises engineered endogenous peptides and macrocycles containing various protraction methods and noncanonical amino acids in assay conditions at varying pH. The model was trained with the pH appended to the model embedding prior to the regression head, with all weights tuned.

**Fig. 5:**
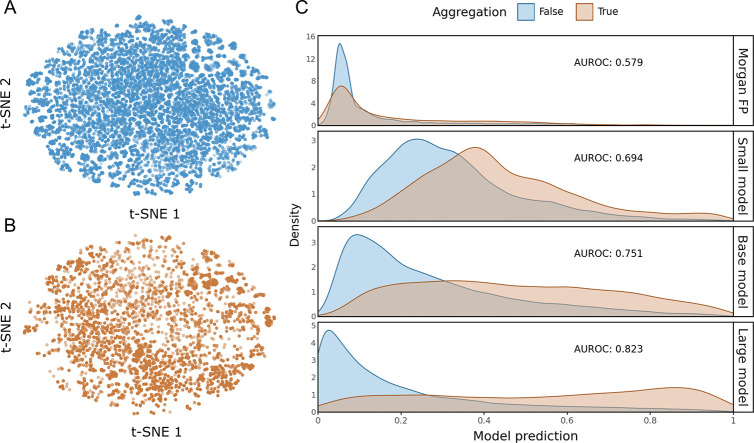
Impact of model capacity on classification of peptide aggregation. The t-SNE visualization of the embedding space shows significant overlap between (A) negative aggregation and (B) positive aggregation. (C) Density plots of model predictions for non-aggregating vs. aggregating peptides. Conventional molecular fingerprints (Morgan FP) fail to distinguish the two populations (AUROC 0.579). In contrast, increasing PeptideMTR model size yields improvement in class separation, reaching an AUROC of 0.823 at 337M parameters. Each curve is scaled independently with AUC = 1.0

Embeddings from the MLM-MTR model show that there is minimal separation in learned features and descriptor space, making this a harder classification problem (Fig. 5A&B). A Random Forest regressor trained on Morgan Fingerprints failed to discriminate between stable and aggregating peptides, yielding an AUROC of 0.579, performance near random chance, while PeptideMTR demonstrates a distinct scaling law where predictive power is a direct function of model capacity (Fig. 5C).

The Small (32M) and Base (114M) models provided progressive improvements in discrimination, achieving AUROCs of 0.694 and 0.751, respectively. The Large (337M) model achieved the strongest separation, effectively disentangling the aggregating and non-aggregating distributions (AUROC 0.823).

This progression suggests that large-scale chemical language models capture the subtle, non-linear biophysical drivers of aggregation, such as amphipathicity and secondary structure propensity, that are absent in static fingerprints. By enabling the accurate *in silico* identification of aggregation-prone candidates, PeptideMTR provides a rigorous method for de-risking lead candidates early in the drug discovery pipeline and guiding selection of soluble candidates.

## 3 Discussion

In the age of machine learning, the development of therapeutic peptides faces a representational dilemma: protein language models cannot handle non-canonical chemistry, while standard molecular fingerprints lack the contextual depth to model long biopolymers. In our first exploration of this problem space, we developed a SMILES-based model, PeptideCLM [38], which was evaluated by cross-fold prediction of membrane diffusion of cyclic peptides. The work presented here incorporates improvements in data, architecture, scale, and evaluation compared to PeptideCLM. The result is a benchmarked suite of SMILES-based encoder models which we refer to as PeptideMTR. We identified two key factors for effective peptide modeling: a scaling law governing the emergence of chemical priors, and the critical role of specialized tokenization for non-natural residues. Our approach avoids the cost of descriptor-based pretraining and compresses SMILES strings, enabling efficient application of self-attention to complex peptide structures. We also observed that transformer-based encoder models outperform classical fingerprint embeddings when the entire transformer architecture is finetuned on a task.

### 3.1 The scaling transition of inductive bias

Our results demonstrate that the optimal pretraining objective is a function of model capacity. At the 32M parameter scale, explicit physicochemical supervision provides a decisive advantage, effectively guiding the model toward thermodynamic properties that it lacks the capacity to infer from raw syntax alone. This supervisory signal creates a more structured embedding space where permeability is more accurately predicted.

However, this advantage vanishes at scale. The convergence of self-supervised and descriptor-guided models at 337M parameters suggests that sufficiently large transformers can capture these relevant chemical features solely through the statistics of token co-occurrence. This finding offers a mechanistic explanation for prior observations in the field, such as the superiority of descriptor-guided training over standard masked language modeling in ChemBERTa-2 [33]. We posit that ChemBERTa-2, being a relatively lightweight architecture, operated on the left side of this scaling curve, where inductive bias is beneficial. PeptideMTR demonstrates that with sufficient depth, the model becomes less dependent on this auxiliary signal, developing learned representations that are functionally equivalent to those derived from explicit supervision.

### 3.2 Bridge between chemical syntax and biological function

Notably, PeptideMTR is able to generalize beyond physicochemical properties to predict complex biological phenotypes, leveraging the unique advantage of SMILES-based models: the ability to represent any modified residue through its constituent chemical parts. While protein language models are limited to the 20 canonical amino acids, our k-mer SMILES tokenization allows PeptideMTR to efficiently process the key chemical modifications used in peptide therapeutics: cyclization, stereochemical inversions, non-canonical amino acids, and diverse synthetic conjugations.

This capability enabled state-of-the-art performance on AMP and CellPPD-Mod benchmarks, where chemically modified residues frequently break standard predictors. Because the model understands chemical parts, it successfully generalized to a holdout set comprised of noncanonical amino acids, predicting activity for modified residues it has not encountered before. The fact that embeddings pretrained on chemical syntax (SMILES reconstruction) transfer effectively to functional tasks (tumor homing, antimicrobial activity) implies that the grammar of chemical language is fundamentally aligned with the functional logic of biological interactions.

### 3.3 Limitations and future directions

While PeptideMTR establishes a robust baseline for discriminative tasks, several frontiers remain. First, while our results on aggregation (ThT assay) are promising, true *ab-initio* prediction of fibrillation kinetics likely requires capturing dynamic conformational ensembles, which static embeddings may only approximate. Future work could integrate molecular dynamics trajectories into the pretraining corpus to explicitly model flexibility.

Second, our evaluation relied on SMILES strings, which encode topology but not explicit 3D geometry. While the model infers structural constraints (as evidenced by its permeability predictions), integrating geometric deep learning or equivalent 3D-aware pretraining could further enhance performance on structure-dependent targets.

Finally, the scaling transition observed here suggests that we have not yet hit the ceiling of performance. As peptide datasets grow, extending this architecture to the billion-parameter scale could improve predictive ability of the model. High-throughput methods for detection of peptide properties [54] will generate large quantities of ML-ready data, while methods for the synthesis of modified peptides continue to develop [55]. With these data, synthesis methods, and integration of predictive models like PeptideMTR with generative networks [56] (i.e., gradient flow networks or diffusion models), we may soon see the *de-novo* design of non-canonical peptides with precise multi-parametric profiles.

### 3.4 Conclusion

We present PeptideMTR as an open, scalable resource to accelerate the shift from trial-and-error screening to rational design in peptide drug discovery. By resolving the trade-off between model capacity and chemical interpretability, PeptideMTR provides the community with a unified framework for engineering the next generation of stable, potent, and chemically diverse therapeutics. We release all model weights, tokenizers, and datasets to foster reproducibility and innovation in this critical domain.

## 4 Methods

### 4.1 Dataset curation and preprocessing

To construct a chemically diverse pretraining corpus bridging the gap between small molecules and proteins, we curated data from three primary sources: PubChem [45], ESMAtlas [18], and the LIPID MAPS Structure Database (LMSD) [44].

#### Small molecules:

We retrieved the complete compound set from PubChem and applied a rigorous filtering cascade to remove non-drug-like entities. Entries were excluded if they were shorter than 20 characters or contained silicon chains. We further removed salts (leading/trailing Br and Cl) and disconnected components (e.g., solvent molecules indicated by ‘.’ within brackets). Polymeric artifacts, specifically repeating silicon oxide motifs (e.g., [Si](=O)[Si](=O)), were identified and discarded. After splitting remaining disconnected components into independent entries and deduplicating the dataset, the final small-molecule corpus contained 108,583,157 unique SMILES strings.

#### Peptides:

Peptide sequences were sourced from ESMAtlas. To ensure high-quality structural priors, we filtered for sequences with high predicted confidence (pTM > 0.7, pLDDT > 0.7) and lengths ≤ 100 amino acids. To reduce redundancy, sequences were clustered at 30% identity using MMseqs2 (--min-seq-id 0.3 -c 0.8 --cov-mode 1), retaining the centroid with the highest product of pTM and pLDDT as the cluster representative.

#### Lipids:

All available lipid structures were obtained directly from LMSD without downsampling.

#### Data Balancing:

To prevent model collapse into the dominant small-molecule modality, we employed a balanced sampling strategy during training. Each epoch consisted of an upsampled lipid set (250k), the full peptide set (~10M), and a random downsampled subset of small molecules (10M), ensuring the model encountered a heterogeneous distribution of chemical syntax. All molecules were canonicalized to standard SMILES format using RDKit; entries failing conversion were discarded.

### 4.2 K-mer SMILES tokenization

Standard atom-level tokenization often results in excessively long sequences for peptide biopolymers, increasing the computational cost of self-attention (O(*n*^2^)). To mitigate this, we developed a custom k-mer tokenizer based on the concepts from SMILES Pair Encoding [57] that compresses peptide SMILES while preserving chemical semantics.

We first constructed a pre-tokenizer that segments SMILES into atom-level primitives, preserving multi-character elements (e.g., ”Br”, ”Cl”) and stereochemical/charge brackets (e.g., [C@@H], [N+]). We then mined the most frequent contiguous k-mers (up to length 6) from a reference corpus of 200,000 PubChem small molecules and 200,000 SmProt peptides [58]. This candidate list was filtered based on chemical validity and frequency (Tab. S2), yielding a compact vocabulary of 405 tokens (160 single-atom, 245 k-mer). This vocabulary reduces sequence length by approximately 60% compared to character-level encoding, enabling efficient training on longer peptide chains.

### 4.3 Pretraining objectives

#### 4.3.1 Masking distributions

The masking procedure employs a Gaussian distribution to determine the lengths of token spans to be masked within sequences. Each sequence’s length is used to calculate the total number of tokens to mask, based on a specified masking percentage.

For each sequence, the procedure samples span lengths with a mean of 3.5 and a standard deviation of 1.0, ensuring a minimum span of 1 token. A random starting position is selected for each span, and the end position is adjusted to prevent exceeding the sequence length. The process includes checking for overlapping positions to avoid masking the same token multiple times.

Masked positions are updated with a specified mask token ID, and the operation continues until the desired number of tokens is masked across all sequences.

### 4.4 Model architecture and pretraining

#### 4.4.1 Architecture

PeptideMTR models are trained as BERT-style transformer encoders [37]. We instantiated three model scales:

**Small (32M):** 6 layers, 384 hidden dimension, 6 heads.

**Base (114M):** 12 layers, 768 hidden dimension, 12 heads.

**Large (337M):** 24 layers, 1024 hidden dimension, 16 heads.

All models utilize Rotary Positional Embeddings (RoPE) to better capture relative distances in chemical space, along with SwiGLU activation functions and pre-layer normalization for training stability. Full hyperparameters can be found in Table S1.

#### 4.4.2 Training objectives

We employed a hybrid objective function combining self-supervision with property regression:

$$ℒ=\lambda_{MLM}\cdot {ℒ}_{MLM}+\lambda_{MTR}\cdot {ℒ}_{MTR}$$

##### Masked Language Modeling (MLM):

We applied a 25% masking rate using a span-masking strategy to encourage the reconstruction of chemical fragments rather than trivial atoms. Span lengths were sampled from a Gaussian distribution (μ=3.5,σ=1), with each masked position replaced by the [MASK] token.

##### Multi-Task Regression (MTR):

In parallel, a regression head predicted 99 physicochemical descriptors (computed via RDKit) from the mean-pooled sequence embedding. The MTR head consists of two fully connected layers with SiLU activation [41]. Descriptors were normalized to zero mean and unit variance prior to training.

For the combined objective, we set $\lambda_{MLM}=0.6$ and $\lambda_{MTR}=0.4$. To enforce structural invariance, we applied dynamic randomization of SMILES strings during training [59], ensuring the model sees different valid string representations of the same molecule.

#### 4.4.3 Optimization

Models were trained using the AdamW optimizer ($\beta_{1}=0.9,\;\beta_{2}=0.98$, weight decay 0.01). Pretraining was performed on 8× NVIDIA H100 GPUs with a global batch size of 512 for 3 pseudo-epochs. Each pseudo-epoch consisted of a sample of ~20.25M molecules, comprising 10M small molecules, the full ESMAtlas, and a 5× upsampling of the LMSD. The learning rate followed a linear warmup for the first 5,000 steps to a peak of 3 × 10^−4^, followed by cosine annealing to 10% of the peak rate.

### 4.5 Downstream evaluation protocols

#### 4.5.1 Fine-tuning strategy

For all downstream tasks, we replaced the pretraining heads with a task-specific feedforward network consisting of two hidden layers with GeLU activation and dropout (*p* = 0.1). Optimization was performed using AdamW with a learning rate of 1 × 10^−5^ and a batch size of 16 unless otherwise noted.

Train/test splitting was strictly replicated from the benchmark literature for each dataset to ensure fair comparison. For aggregation prediction, we performed stratified 5-fold cross-validation with random splits.

Models were trained until validation loss plateaued (patience of 5 epochs, checking validation every 0.5 epoch). To improve robustness, final predictions are reported as the ensemble mean of models trained on the inner cross-validation folds.

#### 4.5.2 Classification Baselines

As a primary baseline in classification tasks, we computed RDKit topological fingerprints (2048-bit, path length 1–7, 2 bits/hash) [40] and trained Random Forest classifiers using scikit-learn [60]. These models utilized 100 estimators (n_estimators=100) with the standard Gini impurity criterion. Tree depth was unconstrained, allowing nodes to expand until all leaves were pure. For specific tasks, we compared against published specialized architectures including AmpHGT [52] and THPep [50].

#### 4.5.3 Layer-wise transfer learning, CycPeptMPDB

To assess the intrinsic quality of learned representations without weight updates, we froze the pretrained encoder and extracted embeddings from each layer. These embeddings were mean-pooled over the sequence length and passed to a LassoCV regressor (5-fold cross-validation). We allowed the model to automatically select the regularization parameter (*α*) over a path of 100 values with a maximum of 10,000 iterations.

## Supplementary Material

Supplement 1

Supplementary tables and figures are included in this publication.

## Data Availability

Pretraining data has been released on Zenodo (doi:10.5281/zenodo.17993164) and on Huggingface datasets in the PeptideMTR collection for ease of use. Aside from the proprietary fibrillation dataset from Novo Nordisk, all data for finetuning can be found on the project github.

## Code availability

All code for pretraining, finetuning, and data processing for this project has been made available at https://github.com/AaronFeller/PeptideMTR.

## Model availability

Model code and weights for all models are released on huggingface at https://huggingface.co/collections/aaronfeller/peptidemtr.

## References

[1] Muttenthaler M., King G. F., Adams D. J. & Alewood P. F. Trends in peptide drug discovery. Nat. Rev. Drug Disc. 20, 309–325 (2021). 10.1038/s41573-020-00135-8 33536635

[2] Cooper B. M., Iegre J., O’Donovan D. H., Halvarsson M. Ö. & Spring D. R. Peptides as a platform for targeted therapeutics for cancer: peptide–drug conjugates (PDCs). Chem. Soc. Rev. 50, 1480–1494 (2021). 33346298 10.1039/d0cs00556h

[3] Wang L. Therapeutic peptides: current applications and future directions. Signal Transduct. 7, 48 (2022). 10.1038/s41392-022-00904-4 PMC8844085 35165272

[4] Fetse J., Kandel S., Mamani U.-F. & Cheng K. Recent advances in the development of therapeutic peptides. Trends Pharmacol. Sci. 44, 425–441 (2023). 37246037 10.1016/j.tips.2023.04.003 PMC10330351

[5] Barman P. Strategic approaches to improvise peptide drugs as next generation therapeutics. Int. J. Pept. Res. Ther. 29, 61 (2023). 37251528 10.1007/s10989-023-10524-3 PMC10206374

[6] Sharma K., Sharma K. K., Sharma A. & Jain R. Peptide-based drug discovery: Current status and recent advances. Drug Discov. Today 28, 103464 (2023). 36481586 10.1016/j.drudis.2022.103464

[7] Hickey J. L., Sindhikara D., Zultanski S. L. & Schultz D. M. Beyond 20 in the 21st century: prospects and challenges of non-canonical amino acids in peptide drug discovery. ACS Med. Chem. Lett. 14, 557–565 (2023). 37197469 10.1021/acsmedchemlett.3c00037 PMC10184154

[8] Zhang H. & Chen S. Cyclic peptide drugs approved in the last two decades (2001–2021). RSC Chem. Biol. 3, 18–31 (2022). 35128405 10.1039/d1cb00154j PMC8729179

[9] Ji X., Nielsen A. L. & Heinis C. Cyclic peptides for drug development. Angew. Chem. Int. Ed. 202308251, e202308251 (2023). 10.1002/anie.202308251 37870189

[10] Lamers C. Overcoming the shortcomings of peptide-based therapeutics. Future Drug Discov. 4, FDD75 (2022).

[11] Openy J. Backbone alterations in cyclic peptides influence both membrane permeability and biological activity. J. Med. Chem. 68, 24108–24126 (2025). 41196074 10.1021/acs.jmedchem.5c01901 PMC12670408

[12] Zhu C. Identification of non-canonical peptides with moPepGen. Nat. Biotechnol. 1–6 (2025). 40523945 10.1038/s41587-025-02701-0 PMC12680078

[13] Du Z., Caragea D., Guo X. & Li Y. PepBERT: Lightweight language models for bioactive peptide representation. bioRxiv 10.1101/2025.04.08.647838 (2025).

[14] Wang L. PepDoRA: A unified peptide language model via weight-decomposed low-rank adaptation. *arXiv* 2410.20667 (2024).

[15] Fernández-Díaz R., Ochoa R., Hoang T. L., Lopez V. & Shields D. How to build machine learning models able to extrapolate from standard to modified peptides. J. Cheminform. (2025). 10.1186/s13321-025-01115-z PMC12751563 41310881

[16] Consortium T. U. UniProt: the universal protein knowledgebase in 2025. Nucleic Acids Res. 53, D609–D617 (2025). 39552041 10.1093/nar/gkae1010 PMC11701636

[17] Rives A. Biological structure and function emerge from scaling unsupervised learning to 250 million protein sequences. Proc. Natl. Acad. Sci. 118, e2016239118 (2021). 33876751 10.1073/pnas.2016239118 PMC8053943

[18] Lin Z. Evolutionary-scale prediction of atomic-level protein structure with a language model. Science 379, 1123–1130 (2023). 36927031 10.1126/science.ade2574

[19] Hayes T. Simulating 500 million years of evolution with a language model. Science 387, 850–858 (2025). 39818825 10.1126/science.ads0018

[20] Elnaggar A. ProtTrans: towards cracking the language of life’s code through self-supervised learning. IEEE Trans. Pattern Anal. Mach. Intell. 44, 7112–7127 (2021). 10.1109/TPAMI.2021.3095381 34232869

[21] Alanazi W., Meng D. & Pollastri G. Porter 6: protein secondary structure prediction by leveraging pre-trained language models (PLMs). Int. J. Mol. Sci. 26, 130 (2024). 39795988 10.3390/ijms26010130 PMC11719765

[22] Zhang Z. Protein language models learn evolutionary statistics of interacting sequence motifs. Proc. Natl. Acad. Sci. 121, e2406285121 (2024). 39467119 10.1073/pnas.2406285121 PMC11551344

[23] Heinzinger M. Contrastive learning on protein embeddings enlightens midnight zone. NAR Genom. Bioinform. 4, lqac043 (2022). 35702380 10.1093/nargab/lqac043 PMC9188115

[24] Sun Y. & Shen Y. Structure-informed protein language models are robust predictors for variant effects. Hum. Genet. 144, 209–225 (2025). 39117802 10.1007/s00439-024-02695-w PMC12068927

[25] Gurev S., Youssef N., Jain N. & Marks D. Variant effect prediction with reliability estimation across priority viruses. bioRxiv 10.1101/2025.08.04.668549 (2025).

[26] Liu D. PLM-interact: extending protein language models to predict protein-protein interactions. Nat. Commun. 16, 9012 (2025). 41145424 10.1038/s41467-025-64512-w PMC12559430

[27] Lu A. X., Zhang H., Ghassemi M. & Moses A. Self-supervised contrastive learning of protein representations by mutual information maximization. BioRxiv 10.1101/2020.09.04.283929 (2020).

[28] Weininger D. SMILES, a chemical language and information system. J. Chem. Inf. Comput. Sci. 28, 31–36 (1988).

[29] Krenn M. SELFIES and the future of molecular string representations. Patterns 3, 100588 (2022). 36277819 10.1016/j.patter.2022.100588 PMC9583042

[30] Wang S., Guo Y., Wang Y., Sun H. & Huang J. SMILES-BERT: Large-scale unsupervised pre-training for molecular property prediction. Proc. ACM Conf. Bioinform. Comput. Biol. 429–436 (2019).

[31] Chithrananda S., Grand G. & Ramsundar B. ChemBERTa: large-scale self-supervised pretraining for molecular property prediction. arXiv 2010.09885 (2020).

[32] Ross J. Large-scale chemical language representations capture molecular structure and properties. Nat. Mach. Intell. 4, 1256–1264 (2022).

[33] Ahmad W., Simon E., Chithrananda S., Grand G. & Ramsundar B. ChemBERTa-2: Towards chemical foundation models. *arXiv* 2209.01712 (2022).

[34] Lv L. ProLLaMA: A protein large language model for multi-task protein language processing. IEEE Trans. Artif. Intell. (2025).

[35] Praski M., Adamczyk J. & Czech W. Benchmarking pretrained molecular embedding models for molecular representation learning. *arXiv* 2508.06199 (2025).

[36] Adamczyk J., Ludynia P. & Czech W. Molecular fingerprints are strong models for peptide function prediction. *arXiv* 2501.17901 (2025). 10.1093/bioinformatics/btag179 PMC13143419 41981726

[37] Devlin J., Chang M.-W., Lee K. & Toutanova K. BERT: Pre-training of deep bidirectional transformers for language understanding. Proc. NAACL 4171–4186 (2019).

[38] Feller A. L. & Wilke C. O. Peptide-aware chemical language model successfully predicts membrane diffusion of cyclic peptides. J. Chem. Inf. Model. 65, 571–579 (2025). 39772542 10.1021/acs.jcim.4c01441 PMC11971985

[39] Borchani H., Varando G., Bielza C. & Larranaga P. A survey on multi-output regression. WIREs Data Min. Knowl. Discov. 5, 216–233 (2015).

[40] Landrum G. RDKit: Open-source cheminformatics (2013). URL http://www.rdkit.org. Online.

[41] Elfwing S., Uchibe E. & Doya K. Sigmoid-weighted linear units for neural network function approximation in reinforcement learning. Neural Netw. 107, 3–11 (2018). 29395652 10.1016/j.neunet.2017.12.012

[42] Xiong R. On layer normalization in the transformer architecture. Proc. Int. Conf. Mach. Learn. (ICML) 10524–10533 (2020).

[43] Joshi M. SpanBERT: Improving pre-training by representing and predicting spans. Trans. Assoc. Comput. Linguist. 8, 64–77 (2020).

[44] Sud M. LMSD: LIPID MAPS structure database. Nucleic Acids Res. 35, D527–D532 (2007). 17098933 10.1093/nar/gkl838 PMC1669719

[45] Kim S. PubChem 2025 update. Nucleic Acids Res. 53, D1516–D1525 (2025). 39558165 10.1093/nar/gkae1059 PMC11701573

[46] Morgan H. L. The generation of a unique machine description for chemical structures-a technique developed at Chemical Abstracts Service. J. Chem. Doc. 5, 107–113 (1965).

[47] Ramsundar B. Deep Learning for the Life Sciences (O’Reilly, 2019).

[48] Li J. CycPeptMPDB: a comprehensive database of membrane permeability of cyclic peptides. J. Chem. Inf. Model. 63, 2240–2250 (2023). 36930969 10.1021/acs.jcim.2c01573 PMC10091415

[49] Scodeller P. & Asciutto E. K. Targeting tumors using peptides. Molecules 25, 808 (2020). 32069856 10.3390/molecules25040808 PMC7070747

[50] Shoombuatong W., Schaduangrat N., Pratiwi R. & Nantasenamat C. THPep: A machine learning-based approach for predicting tumor homing peptides. Comput. Biol. Chem. 80, 441–451 (2019). 31151025 10.1016/j.compbiolchem.2019.05.008

[51] Kumar V. Prediction of cell-penetrating potential of modified peptides containing natural and chemically modified residues. Front. Microbiol. 9, 725 (2018). 29706944 10.3389/fmicb.2018.00725 PMC5906597

[52] He Y., Song X., Wan H. & Zhao X. AmpHGT: expanding prediction of antimicrobial activity in peptides containing non-canonical amino acids using multi-view constrained heterogeneous graph transformer. BMC Biol. 23, 184 (2025). 40598389 10.1186/s12915-025-02253-4 PMC12217533

[53] Xue C., Lin T. Y., Chang D. & Guo Z. Thioflavin T as an amyloid dye: fibril quantification, optimal concentration and effect on aggregation. R. Soc. Open Sci. 4, 160696 (2017). 28280572 10.1098/rsos.160696 PMC5319338

[54] Quartararo A. J. Ultra-large chemical libraries for the discovery of high-affinity peptide binders. Nat. Commun. 11, 3183 (2020). 32576815 10.1038/s41467-020-16920-3 PMC7311396

[55] Niquille D. L. Nonribosomal biosynthesis of backbone-modified peptides. Nat. Chem. 10, 282–287 (2018). 29461527 10.1038/nchem.2891

[56] Shin J.-E. Protein design and variant prediction using autoregressive generative models. Nat. Commun. 12, 2403 (2021). 33893299 10.1038/s41467-021-22732-w PMC8065141

[57] Li X. & Fourches D. SMILES pair encoding: a data-driven substructure tokenization algorithm for deep learning. J. Chem. Inf. Model. 61, 1560–1569 (2021). 33715361 10.1021/acs.jcim.0c01127

[58] Li Y. SmProt: a reliable repository with comprehensive annotation of small proteins identified from ribosome profiling. Genomics Proteomics Bioinformatics 19, 602–610 (2021). 34536568 10.1016/j.gpb.2021.09.002 PMC9039559

[59] Arús-Pous J. Randomized SMILES strings improve the quality of molecular generative models. J. Cheminform. 11, 71 (2019). 33430971 10.1186/s13321-019-0393-0 PMC6873550

[60] Pedregosa F. Scikit-learn: Machine learning in Python. J. Mach. Learn. Res. 12, 2825–2830 (2011).

