# Scaling SMILES-Based Chemical Language Models for Therapeutic Peptide Engineering

**DOI:** 10.64898/2026.01.06.697994

**Published:** 2026-02-12

**Authors:** Aaron L. Feller, Maxim Secor, Sebastian Swanson, Claus O. Wilke, Kristine Deibler

**Affiliations:** 1 Molecular AI, Novo Nordisk, 33 Hayden Ave, Lexington, 02421, MA, USA.; 2 Integrative Biology, The University of Texas at Austin, 2500 Speedway, Austin, 78712, TX, USA.

**Keywords:** chemical language models, peptide representation learning, multi-task regression, foundation models, scaling laws, machine learning for drug discovery, noncanonical peptides

## Abstract

Therapeutic peptides occupy a unique middle ground in drug discovery, offering the high specificity of protein interactions with the chemical diversity of small molecules, yet they currently fall in a computational blind spot. Existing AI tools cannot handle them effectively: protein models are restricted to natural amino acids, while chemical models struggle to process large, polymer-like sequences. This disconnect has forced the field to rely on static chemical descriptors that fail to capture subtle chemical details. To bridge this gap, we present PeptideCLM-2, a chemical language model trained on over 100 million molecules to natively represent complex peptide chemistry. PeptideCLM-2 consistently outperforms both chemical descriptors and specialized AI models on critical drug development tasks, including aggregation, membrane diffusion, and cell targeting. Notably, we find that when model parameters reach the 100 million scale, the transformer architecture is able to learn chemical properties from molecular syntax alone.

## 1 Introduction

Peptides are a rapidly evolving modality in the therapeutic space [1–6]. They occupy a unique chemical niche situated between small molecules and proteins. Like small molecules, they possess immense chemical diversity [7–11]. Yet, they retain the modularity of biological polymers, allowing for accessible and efficient synthesis [12–14]. While modern synthetic methods enable the creation of a vast diversity of modified peptides, the computational tools required to rationally select among these options have not kept pace. Attempts to adapt existing frameworks have faced significant limitations. Protein language models (PLMs) are restricted to fixed amino-acid alphabets, rendering them unable to encode noncanonical or chemically modified residues [15]. Conversely, chemical language models (CLMs) are typically trained on small molecules, lacking the contextual range to interpret peptide-specific motifs [16, 17].

Large-scale protein corpora such as UniProt [18] have fueled the rise of self-supervised PLMs. Architectures like the ESM family [19–21] and ProtTrans [22] have proven that transformers can learn structural and functional constraints directly from sequence data. These models capture deep dependencies predictive of structure [23–25], variant effects [26, 27], and molecular function [28, 29]. In parallel, CLMs utilizing symbolic representations like SMILES or SELFIES [30, 31] have successfully encoded chemical syntax via masked or autoregressive objectives [32–34], enabling prediction of various chemical properties [35, 36]. Extensions such as ChemBERTa-2 [37] and ProLLaMA [38] have further suggested that augmenting self-supervision with physicochemical regression introduces a valuable inductive bias, improving generalization. Despite these advances, however, deep learning has historically shown only minimal, if any, improvement over molecular fingerprints for peptide property prediction [39, 40].

In this work, we introduce PeptideCLM-2, a suite of nine SMILES-based transformer encoders [41] designed to unify therapeutic peptide modeling. Building upon our previous architecture, PeptideCLM [42], we train models across a parameter range of 32 to 337 million using three distinct objectives: masked language modeling (MLM), multi-task regression (MTR) [43] to RDKit-derived descriptors [44], and a dual objective combining both. This systematic design provides a framework to rigorously decouple the effects of model scale and inductive bias on representation learning. Because therapeutic peptides often adopt transient rather than static conformations, we employed a string-based architecture to capture topological connectivity without the bias of a single, rigid 3D structure.

We demonstrate that these models outperform molecular fingerprints and specialized architectures, while relying only on simple string-based inputs. Our evaluation also reveals a critical scaling transition: while descriptor-guided pretraining provides a necessary scaffold for smaller models, larger architectures trained solely on MLM spontaneously recover these physicochemical relationships, a capability evident in both the embedding manifold and downstream evaluations. Together, these results establish PeptideCLM-2 as both an open, scalable resource for peptide engineering and a framework for understanding the interaction between model capacity and pretraining for the field of computational peptide chemistry.

## 2 Results

### 2.1 Model architecture and training design

The PeptideCLM-2 suite represents a unified framework for decoding the language of therapeutic peptides. Unlike standard protein language models, which are restricted to a fixed alphabet of 20 canonical amino acids, PeptideCLM-2 is designed to explore the full spectrum of chemical space. By combining scalable transformer architectures with interpretable tokenization strategies, the framework provides a bridge between symbolic chemical representation and chemical function.

To rigorously evaluate chemical deep learning for peptides and the emergence of chemical intuition, we designed a controlled grid of nine models. We varied model capacity across three orders of magnitude—32M, 114M, and 337M parameters—and trained each scale using three distinct pretraining objectives: masked-language modeling (MLM), multi-task regression (MTR) to physicochemical descriptors, and a combined dual objective. This systematic design allows us to disentangle the effects of parameter scale from the learning paradigm used.

**Fig. 1:**
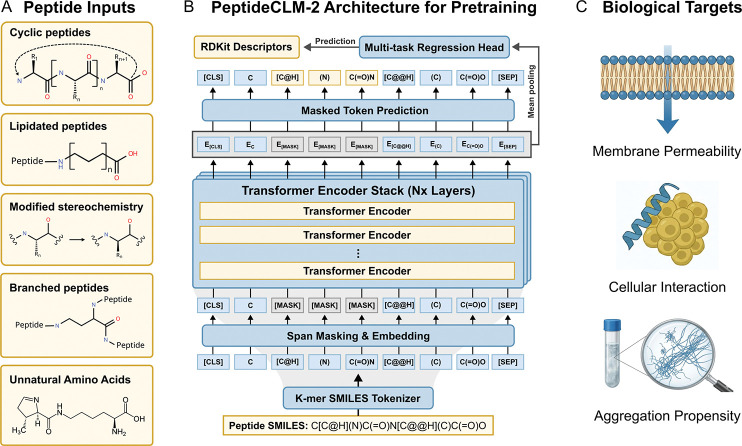
PeptideCLM-2 workflow: enabling encoding of chemical diversity for predictive modeling. (A) The model can input SMILES strings from peptides with various modifications, allowing it to encode cyclic peptides, non-canonical amino acids, and synthetic modifications. (B) Models were trained with both span masking (predicting grey [MASK] tokens) and regression to physicochemical descriptors (e.g., LogP, TPSA) from a pooled sequence embedding. (C) Examples of prediction targets for downstream tasks, including membrane permeability, cellular interactions, and aggregation propensity.

Our architecture processes inputs as raw SMILES strings. This allows for the native encoding of canonical residues, non-canonical modifications, cyclic scaffolds, and complex conjugations like lipidation or PEGylation (Fig. 1A). The backbone of the framework is a BERT-style transformer encoder (Fig. 1B). To ensure training stability and effective handling of long-range chemical dependencies within large macrocycles, we incorporated modern architectural features including rotary positional embeddings (RoPE) [45], SwiGLU activation functions [46], and pre-layer normalization [47]. Model depth and hidden dimension scale with parameter count, maintaining a consistent per-head dimensionality of 64.

We investigated three distinct learning paradigms to train the architecture. The Masked Language Modeling (MLM) objective utilized span masking [48] to force the model to reconstruct missing chemical fragments, effectively learning the syntax of molecular graphs. The Multi-Task Regression (MTR) objective explicitly grounded the embeddings by regressing to 99 RDKit-derived physicochemical descriptors (Tab. S1) from a mean-pooled embedding. Finally, the Dual Objective (MLM-MTR) optimized both losses simultaneously, testing whether syntax and semantics could be learned in tandem. For downstream evaluation, the pretrained encoder is coupled with a lightweight, 2-hidden layer feed-forward regression head employing GeLU activation, allowing the model to adapt its learned representations to specific biological tasks such as membrane permeability, cellular interactions, or aggregation (Fig. 1C).

### 2.2 Pretraining data and tokenization schema

**Fig. 2:**
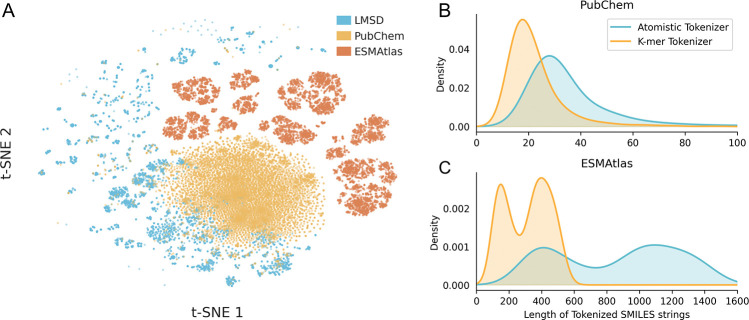
Data Diversity and Representational Efficiency. (A) A 2D t-SNE projection of Morgan Fingerprints from the pretraining corpus, demonstrating that the composite dataset (LMSD, PubChem, and ESMAtlas) bridges distinct chemical subspaces. (B) Comparative length distributions of tokenized molecules. The k-mer tokenization strategy yields a substantial compression factor relative to atom-level encoding, reducing sequence length by 64% for peptides, thereby enabling the efficient processing of long biological chains.

To construct a representation space that spans the full continuum from small molecules to biological polymers, we curated a composite pretraining corpus combining three distinct datasets. We aggregated lipids from the LIPID MAPS Structure Database (*n* = 50,450) [49], small drug-like molecules from PubChem (*n* = 108,583,157) [50], and diverse peptide sequences from ESMAtlas (*n* = 9,634,945) [20]. As visualized in Figure 2A, these sources inhabit unique, complementary regions of the chemical manifold, ensuring the model encounters a heterogeneous distribution of molecular syntax [51]. Due to the flexibility of SMILES encoding, we are able to train on small molecules to learn chemical features, which can directly translate to therapeutic peptides that contain chemistries not seen in the natural amino acid space.

A central bottleneck in applying chemical language models to biopolymers is the computational cost of self-attention, which scales quadratically with sequence length $\left(𝒪\left(n^{2}\right)\right)$. Because direct character encoding of peptides generates exceptionally long SMILES strings, standard models become prohibitively expensive to train. To resolve this, we developed a specialized k-mer tokenizer (Tab. S2 & S3) that compresses these symbolic representations by mapping recurring sub-structural motifs to single tokens. This strategy significantly reduces the effective sequence length, and thus the quadratic compute burden, while maintaining full compatibility with standard SMILES syntax.

Quantitatively, this strategy reduces sequence lengths by 38% for small molecules and 64% for natural peptides relative to standard atom-level frameworks like DeepChem [52] (Fig. 2B). We found that this compression comes at no cost to accuracy; benchmarking confirms that models trained with either tokenizer achieve equivalent performance on membrane permeability prediction (Table S8). Thus, the k-mer approach successfully resolves the trade-off between computational tractability and semantic fidelity, allowing the model to attend to long-range dependencies in complex backbones without the prohibitive cost of character-level encoding.

To leverage this efficient representation for rigorous benchmarking, we held the data composition and tokenization schema constant across all experiments (Tab. S4). We similarly standardized finetuning workflows using nested cross-validation (Tab. S5), ensuring that observed differences in downstream tasks, such as permeability or aggregation, are attributable solely to the emergence of learned chemical intuition rather than artifacts from pretraining.

### 2.3 The scaling transition of physicochemical intuition

We evaluated PeptideCLM-2 using the CycPeptMPDB dataset [53], a standard benchmark for cyclic peptide permeability with robust evaluation splits [42]. To ensure a rigorous comparison, we focused our analysis on nine distinct model configurations selected via ablation studies (Tab. S6), covering three distinct parameter sizes.

**Fig. 3:**
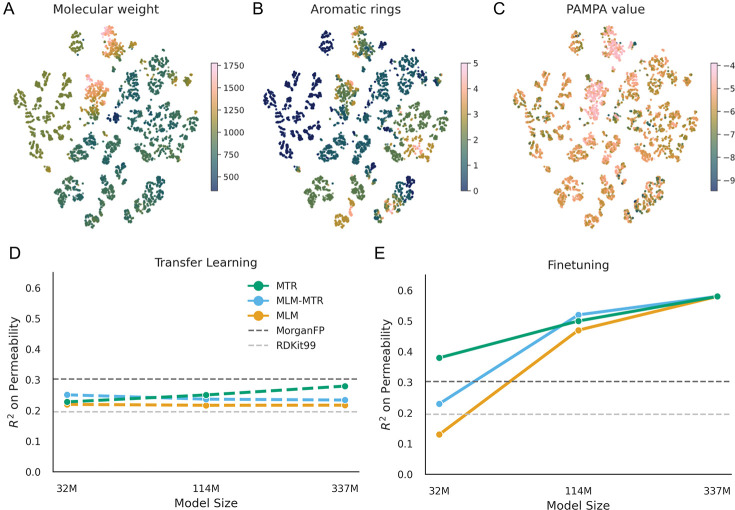
Emergence of chemical structure and predictive capability. (A-C) A t-SNE projection of embeddings from the 337M model. The model spontaneously organizes the chemical manifold by (A) molecular weight and (B) aromaticity, which correlates with (C) measured permeability. (D) Transfer learning performance (linear probing) remains low across all scales, indicating permeability requires non-linear features. (E) Full finetuning reveals a scaling transition: while small models rely on explicit supervision (MTR) to perform well, large self-supervised models (MLM) spontaneously recover this performance, matching the supervised baseline.

We first investigated whether the models had learned a chemically meaningful latent space. By projecting the embeddings of the 337M MLM model into two dimensions, we observed that the model organizes the chemical manifold according to fundamental physical properties. Without explicit instruction, MLM training organized embeddings by molecular weight and aromaticity (Fig. 3A, B). We also notice this across a larger list of physicochemical descriptors including charge, logP, and available hydrogen bonding sites (Fig. S1). This unsupervised organization shows that the embedded structure also has some organization with measured PAMPA permeability (Fig. 3C), confirming that the model has encoded many of the structural determinants of membrane diffusion.

To disentangle the quality of these raw representations from the model’s capacity to adapt, we compared layer-wise transfer learning against full-model finetuning. We found that linear probes trained on frozen features performed poorly (*R*^2^ *<* 0.3) regardless of model scale (Fig. 3D), suggesting that the model embeddings are complex, non-linear properties that cannot be extracted via simple linear combinations of features.

We further analyzed *where* this information is stored by probing each layer of the network. This revealed a significant difference in stability between model sizes. In smaller models, the final hidden layer—typically used for downstream tasks—often underperformed compared to intermediate layers (Fig. S2), suggesting a ”forgetting” of physicochemical details at the output bottleneck. In contrast, larger models exhibited high stability, maintaining rich representations from the middle layers all the way to the final output.

The most significant finding, however, emerged during full-model finetuning (Fig. 3E). At the smallest scale (32M), inductive bias is dominant: models pretrained with explicit physicochemical regression (MTR) significantly outperformed those trained only on language modeling (*R*^2^ ≈ 0.38 vs. 0.13). This indicates that small models improve when pretraining is grounded in physical reality.

As capacity increased to 337M parameters, this dependency vanished. The purely self-supervised MLM models show equivalent predictions to the supervised MTR models, with both objectives converging at an *R*^2^ ≈ 0.58. This is nearly double the performance of traditional molecular fingerprints (*R*^2^ ≈ 0.3). Here we demonstrate a fundamental scaling law: while small models improve prediction when explicitly taught physical properties of molecules, sufficiently large transformers are able to derive these priors from the syntax of chemical language alone.

### 2.4 Generalization to biological function: Bioactivity and localization

Having established that PeptideCLM-2 captures physicochemical constraints, we next asked whether this intuition translates to complex biological function. We evaluated the model on three diverse classification benchmarks—tumor homing, cell penetration, and antimicrobial activity (Tab. S7)—using Matthews Correlation Coefficient (MCC) to rigorously account for class imbalance. The datasets used for these tasks all require modeling of non-canonical chemistry.

**Fig. 4:**
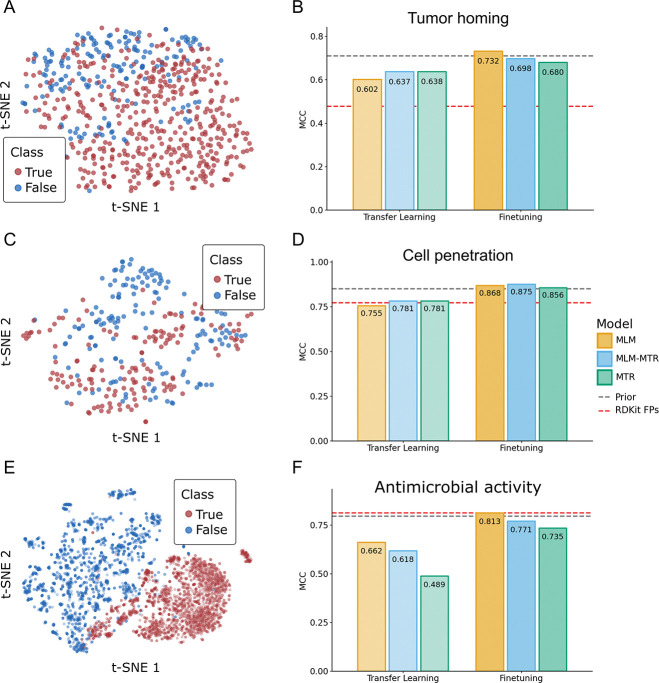
Predictions on three benchmark datasets for biological interaction. **Left panels:** t-SNE projections (A, C, E) of embeddings from the 337M MLM-MTR model. Even before finetuning, the model’s latent space exhibits strong linear separability between active (red) and inactive (blue) peptides across all three tasks. **Right panels:** Performance comparison (MCC) between Transfer Learning (linear probe) and End-to-End Finetuning (B, D, F). Grey dashed lines indicate the performance of prior state-of-the-art methods (THPep, Random Forest+PaDEL, AmpHGT). Red dashed lines indicate a standard Random Forest baseline using RDKit topological fingerprints.

Visual inspection of the pretrained embedding space reveals that the model has already learned to distinguish bioactive species. Projections of the 337M MLM-MTR embeddings show distinct separation between positive and negative classes across all three benchmarks (Fig. 4A, C, E). This latent structure provides a robust foundation for finetuning, where PeptideCLM-2 models were able to outperform specialized architectures, often relying on generation of chemical features or complicated embedding methods.

#### Tumor Homing:

Tumor-Homing Peptides (THPs) are notoriously difficult to classify because they rely on subtle recognition motifs (e.g., RGD, NGR) to target tumor vasculature [54]. The method published with this dataset, THPep, achieved an MCC of ≈ 0.71 by relying on engineered features like Pseudo Amino Acid Composition (PseAAC) [55]. PeptideCLM-2 surpassed this baseline (MCC 0.732) using only raw SMILES strings (Fig. 4B), demonstrating that the model effectively learns motif-driven features without explicit feature engineering.

#### Cell Penetration:

We next evaluated the model on CellPPD-Mod [56], a dataset explicitly containing chemically modified peptides. Standard sequence models fail here, forcing previous baselines to rely on extracted 2D/3D chemical descriptors (e.g., PaDEL) for the side-chain modifications. The use of PeptideCLM-2 allows for encoding of the entire molecule in one pass, and achieved an MCC of 0.875, outperforming the descriptor-based baselines (≈ 0.85) while operating directly on the chemical syntax (Fig. 4D).

#### Antimicrobial Activity:

Finally, we tested the model on a rigorous benchmark by He et al. [57], designed to challenge models by training on natural peptide sequences and making predictions on peptides containing non-canonical amino acids. The specialized baseline, AmpHGT, employs a complex Heterogeneous Graph Transformer to explicitly model atoms and fragments, achieving an MCC of 0.797. PeptideCLM-2 surpassed this graph-based architecture with an MCC of 0.813 (Fig. 4F). This result shows that a SMILES-based transformer can capture the intrinsic chemistry of non-canonical residues as effectively as explicit molecular graphs.

### 2.5 Predicting peptide aggregation and formulation stability

Peptide aggregation represents a critical failure mode in therapeutic development, compromising safety, efficacy, and shelf-life. Unlike simple solubility, aggregation is driven by complex, condition-dependent interactions between lipids, excipients, and peptide secondary structures. To evaluate PeptideCLM-2 in this space, we utilized a large, proprietary dataset of Thioflavin T (ThT) fluorescence assays, the standard benchmark for assessing fibrillation kinetics [58]. This dataset is particularly challenging as it encompasses engineered macrocycles and endogenous peptides containing diverse protraction moieties and non-canonical amino acids, measured across varying pH conditions. We finetuned the model by appending the pH value to the embedding explicitly before the regression head, allowing the network to learn environment-dependent stability.

**Fig. 5:**
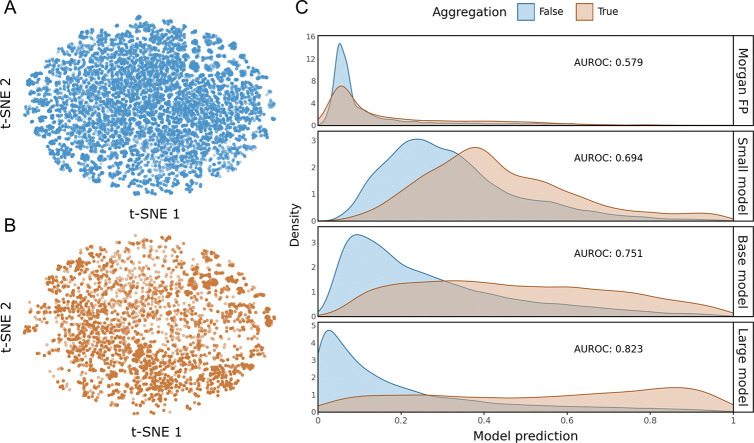
Scaling model size in predicting aggregation. (A-B) t-SNE visualizations of the embedding space show significant overlap between non-aggregating (A) and aggregating (B) peptides, illustrating the difficulty of linearly separating these classes based on structure alone. (C) Density plots of model predictions. Traditional molecular fingerprints (Morgan FP) fail to distinguish the two populations, performing near random chance (AUROC 0.579). In contrast, PeptideCLM-2 exhibits increasing accuracy with model size, reaching an AUROC of 0.823 at 337M parameters. Curves are scaled independently for visibility.

The difficulty of this task is evident in the latent space. Visualizations of the pre-trained embeddings reveal extensive overlap between aggregating and non-aggregating peptides (Fig. 5A&B), indicating that aggregation is not driven by simple structural clusters that are easily separated. Consequently, traditional methods fail completely: a Random Forest trained on Morgan Fingerprints yielded an AUROC of 0.579, performing only slightly better than random chance.

In contrast, PeptideCLM-2 demonstrates a dramatic recovery of predictive power that scales directly with model capacity (Fig. 5C). While the Small (32M) model provided a baseline discrimination (AUROC 0.694), scaling to the Base (114M) and Large (337M) architectures improved performance to 0.751 and 0.823, respectively. This distinct scaling trajectory confirms that large-scale chemical language models are capable of capturing the subtle, non-linear biophysical drivers of aggregation—such as amphipathicity and induced secondary structure—that are invisible to static fingerprints. By accurately identifying fibrillation risks *in silico*, PeptideCLM-2 offers a robust mechanism for de-risking candidates early in the design pipeline, enabling the selection of soluble, stable therapeutics.

## 3 Discussion

The development of therapeutic peptides has long been hindered by a representational dilemma. While machine learning has revolutionized protein engineering, peptides remain stranded between two paradigms: protein language models cannot handle non-canonical chemistry, while standard molecular fingerprints lack the contextual depth to model long biopolymers. Our initial foray into this space, PeptideCLM [42], demonstrated the feasibility of SMILES-based modeling for cyclic peptides. In this work, we expand this concept into a comprehensive framework, PeptideCLM-2, incorporating significant advances in data scale, architectural depth, and rigorous benchmarking that demonstrates that these transformer-based encoders consistently outperform classical fingerprint embeddings.

Our analysis identifies two critical drivers for effective peptide modeling. First, we observe a parameter scaling effect where larger models move beyond simple pattern recognition to spontaneously learn fundamental chemical rules from SMILES notation alone. Second, we introduce a novel k-mer tokenizer to process these complex molecules efficiently. This innovation allows PeptideCLM-2 to analyze intricate structures that were previously too computationally demanding, resolving the conflict between deep chemical accuracy and processing speed.

### 3.1 The scaling transition of inductive bias

Our results demonstrate that the optimal pretraining strategy is strictly a function of model capacity. At the 32M parameter scale, the model operates as a novice: it lacks the capacity to infer thermodynamic laws from raw syntax alone. In this regime, explicit physicochemical supervision provides a decisive advantage, acting as a scaffold that guides the model toward a structured embedding space where permeability can be accurately predicted.

However, this advantage vanishes at scale. The convergence of self-supervised and descriptor-guided models at 337M parameters confirms that sufficiently large transformers outperform chemical features when trained only on token co-occurrence. This finding offers a mechanistic explanation for prior observations in the field, such as the reported superiority of descriptor-guided training in ChemBERTa-2 [37]. We posit that ChemBERTa-2, being a relatively lightweight architecture, operated on the lower end of this scaling curve, where inductive bias is still beneficial, while MoLFormer [34], trained purely on SMILES, operated on the upper end of this curve. PeptideCLM-2 demonstrates that with sufficient depth, the model transcends a dependence on supervised learning, developing learned representations from sequence alone that are functionally equivalent.

### 3.2 Bridge between chemical syntax and biological function

Our reliance on SMILES strings is a deliberate design choice to address the limitations of geometric deep learning in this domain. Unlike globular proteins, which fold into stable tertiary structures, therapeutic peptides are often intrinsically disordered or exist as dynamic ensembles. Current 3D methods force these labile molecules into a single static conformation, introducing a geometric bias that misrepresents their behavior in solution. By operating on SMILES, PeptideCLM-2 captures precise chemical connectivity while implicitly allowing the model to learn representations that account for conformational flexibility. This is supported by our results in membrane permeability, where the model successfully inferred 3D-dependent constraints directly from the 1D syntax without requiring explicit structural input.

Notably, PeptideCLM-2 generalizes beyond simple physicochemical descriptors to predict complex biological phenotypes. This capability stems from the fundamental advantage of SMILES-based architectures: the ability to represent a residue not as a fixed label, but as a composite of its constituent chemical parts. While protein language models are structurally limited to the 20 canonical amino acids, our k-mer tokenization strategy allows PeptideCLM-2 to efficiently process the chemical diversity that defines modern peptide therapeutics, natively encoding cyclizations, stereochemical inversions, and diverse chemical modifications. Additionally, this tokenization method allows the model to learn from small molecule chemistry during pretraining and apply that knowledge on downstream tasks.

This structural flexibility not only enabled simple encoding, but also resulted in improved performance over custom architectures and molecular fingerprints on benchmarks such as AMP and CellPPD-Mod. The model also has demonstrated chemical generalization when successfully predicting the antimicrobial activity of peptides containing non-canonical residues that were held out during training. The finding that embeddings pretrained on chemical syntax (SMILES reconstruction) transfer effectively to functional tasks implies that the chemical features necessary to generalize across molecules are present in the grammar of chemical language.

### 3.3 Limitations and future directions

While PeptideCLM-2 establishes a new standard for discriminative modeling, several frontiers remain. First, peptide aggregation is inherently dynamic. While our static embeddings predict propensity effectively, capturing true fibrillation kinetics may require training on molecular dynamics trajectories to explicitly model conformational flexibility. Second, our reliance on SMILES strings prioritizes topological connectivity over explicit 3D geometry. While the model successfully infers 3D constraints—as evidenced by its permeability predictions—integrating geometric deep learning could further refine performance on highly structure-dependent targets.

As high-throughput screening [59] and advanced synthesis [60] continue to generate large-scale datasets, extending this architecture to the billion-parameter scale promises even greater predictive fidelity. Coupling these predictive oracles with generative frameworks like diffusion models [61] brings us closer to *de novo* design of non-canonical peptides with precise, multi-parametric profiles.

### 3.4 Conclusion

We present PeptideCLM-2 as an open, scalable resource designed to accelerate the transition from empirical screening to rational engineering in peptide drug discovery. By successfully resolving the representational trade-off between semantic depth and computational tractability, our framework empowers the community to design the next generation of stable, potent, and chemically diverse therapeutics. To foster reproducibility and catalyze further innovation, we release all model weights, tokenizers, and training datasets to the public.

## 4 Methods

### 4.1 Dataset curation and preprocessing

To construct a chemically diverse pretraining corpus bridging the gap between small molecules and proteins, we curated data from three primary sources: PubChem [50], ESMAtlas [20], and the LIPID MAPS Structure Database (LMSD) [49].

#### Small molecules:

We retrieved the complete compound set from PubChem and applied a rigorous filtering cascade to remove non-drug-like entities. Entries were excluded if they were shorter than 20 characters or contained silicon chains. We further removed salts (leading/trailing Br and Cl) and disconnected components (e.g., solvent molecules indicated by ‘.’ within brackets). Polymeric artifacts, specifically repeating silicon oxide motifs (e.g., [Si](=O)[Si](=O)), were identified and discarded. After splitting remaining disconnected components into independent entries and deduplicating the dataset, the final small-molecule corpus contained 108,583,157 unique SMILES strings.

#### Peptides:

Peptide sequences were sourced from ESMAtlas. To ensure high-quality structural priors, we filtered for sequences with high predicted confidence (pTM *>* 0.7, pLDDT *>* 0.7) and lengths ≤ 100 amino acids. To reduce redundancy, sequences were clustered at 30% identity using MMseqs2 (--min-seq-id 0.3 -c 0.8 --cov-mode 1), retaining the centroid with the highest product of pTM and pLDDT as the cluster representative. Amino acid sequences were converted to SMILES strings using p2smi [62].

#### Lipids:

All available lipid structures were obtained directly from LMSD.

#### Data Balancing:

To prevent model collapse into the dominant small-molecule modality, we employed a balanced sampling strategy during training. Each epoch consisted of an upsampled lipid set (250k), the full peptide set (~10M), and a random downsampled subset of small molecules (10M), ensuring the model encountered a heterogeneous distribution of chemical syntax. All molecules were canonicalized to standard SMILES format using RDKit; entries failing conversion were discarded.

### 4.2 K-mer SMILES tokenization

Standard atom-level tokenization often results in excessively long sequences for peptide biopolymers, increasing the computational cost of self-attention $\left(𝒪\left(n^{2}\right)\right)$. To mitigate this, we developed a custom k-mer tokenizer based on the concepts from SMILES Pair Encoding [63] that compresses peptide SMILES while preserving chemical semantics.

We first constructed a pre-tokenizer that segments SMILES into atom-level primitives, preserving multi-character elements (e.g., ”Br”, ”Cl”) and stereochemical/charge brackets (e.g., [C@@H], [N+]). We then mined the most frequent contiguous k-mers (up to length 6) from a reference corpus of 200,000 PubChem small molecules and 200,000 SmProt peptides [64]. This candidate list was filtered based on chemical validity and frequency (Tab. S2), yielding a compact vocabulary of 405 tokens (160 single-atom, 245 k-mer). This vocabulary reduces sequence length by approximately 60% compared to character-level encoding, enabling efficient training on longer peptide chains.

### 4.3 Model architecture and pretraining

#### 4.3.1 Architecture

PeptideCLM-2 models are trained as BERT-style transformer encoders [41]. We instantiated three model scales:

##### Small (32M):

6 layers, 384 hidden dimension, 6 heads.

##### Base (114M):

12 layers, 768 hidden dimension, 12 heads.

##### Large (337M):

24 layers, 1024 hidden dimension, 16 heads.

All models utilize Rotary Positional Embeddings (RoPE) to better capture relative distances in chemical space, along with SwiGLU activation functions and pre-layer normalization for training stability. Full hyperparameters can be found in Table S1.

#### 4.3.2 Masking distributions

To implement span masking, we determined span lengths using a Gaussian distribution (*μ* = 3.5, *σ* = 1.0) with a minimum length of one token. The total number of tokens to be masked was calculated per sequence based on a specified masking percentage. For each span, a random starting position was selected, with adjustments made to prevent boundary overruns or overlaps with previously masked regions; selected positions were then replaced with the mask token ID until the target masking budget was satisfied.

#### 4.3.3 Training objectives

We employed a hybrid objective function combining self-supervision with property regression:

$$ℒ=\lambda_{MLM}\cdot {ℒ}_{MLM}+\lambda_{MTR}\cdot {ℒ}_{MTR}$$

##### Masked Language Modeling (MLM):

We applied a 25% masking rate using a span-masking strategy to encourage the reconstruction of chemical fragments rather than trivial atoms. Span lengths were sampled from a Gaussian distribution (*μ* = 3.5, *σ* = 1), with each masked position replaced by the [MASK] token.

##### Multi-Task Regression (MTR):

In parallel, a regression head predicted 99 physicochemical descriptors (computed via RDKit) from the mean-pooled sequence embedding. The MTR head consists of two fully connected layers with SiLU activation [46]. Descriptors were normalized to zero mean and unit variance prior to training.

For the combined objective, we set $\lambda_{MLM}\;=\;0.6$ and $\lambda_{\mathrm{MTR}}\;=\;0.4$. To enforce structural invariance, we applied dynamic randomization of SMILES strings during training [65], ensuring the model sees different valid string representations of the same molecule.

#### 4.3.4 Optimization

Models were trained using the AdamW optimizer (*β*_1_ = 0.9*,β*_2_ = 0.98, weight decay 0.01). Pretraining was performed on 8× NVIDIA H100 GPUs with a global batch size of 512 for 3 pseudo-epochs. Each pseudo-epoch consisted of a sample of ~20.25M molecules, comprising 10M small molecules, the full ESMAtlas, and a 5× upsampling of the LMSD. The learning rate followed a linear warmup for the first 5,000 steps to a peak of 3 × 10^−4^, followed by cosine annealing to 10% of the peak rate.

### 4.4 Downstream evaluation protocols

#### 4.4.1 Finetuning strategy

For all downstream tasks, we replaced the pretraining heads with a task-specific feed-forward network consisting of two hidden layers with GeLU activation and dropout (*p* = 0.1). Optimization was performed using AdamW with a learning rate of 1×10^−5^ and a batch size of 16 unless otherwise noted.

Train/test splitting was strictly replicated from the benchmark literature for each dataset to ensure fair comparison. For aggregation prediction, we performed stratified 5-fold cross-validation with random splits.

Models were trained until validation loss plateaued (patience of 5 epochs, checking validation every 0.5 epoch). To improve robustness, final predictions are reported as the ensemble mean of models trained on the inner cross-validation folds.

#### 4.4.2 Classification Baselines

As a primary baseline in classification tasks, we computed RDKit topological fingerprints (2048-bit, path length 1–7, 2 bits/hash) [44] and trained Random Forest classifiers using scikit-learn [66]. These models utilized 100 estimators (n_estimators=100) with the standard Gini impurity criterion. Tree depth was unconstrained, allowing nodes to expand until all leaves were pure. For specific tasks, we compared against published specialized architectures including AmpHGT [57] and THPep [55].

#### 4.4.3 Layer-wise transfer learning, CycPeptMPDB

To assess the intrinsic quality of learned representations without weight updates, we froze the pretrained encoder and extracted embeddings from each layer. These embeddings were mean-pooled over the sequence length and passed to a LassoCV regressor (5-fold cross-validation). We allowed the model to automatically select the regularization parameter (*α*) over a path of 100 values with a maximum of 10,000 iterations.

## Supplementary Material

Supplement 1

Supplementary information

Supplementary tables and figures are included in this publication.

## Data Availability

Pretraining data has been released on Zenodo (doi:10.5281/zenodo.17993164) and on Huggingface datasets in the PeptideCLM-2 collection for ease of use. Aside from the proprietary fibrillation dataset from Novo Nordisk, all data for finetuning can be found on the project github.

## Code availability

All code for pretraining, finetuning, and data processing for this project has been made available at https://github.com/AaronFeller/PeptideCLM-2.

## Model availability

Model code and weights for all models are released on huggingface at https://huggingface.co/collections/AaronFeller/PeptideCLM-2.

## References

[1] Muttenthaler M., King G. F., Adams D. J. & Alewood P. F. Trends in peptide drug discovery. Nat. Rev. Drug Disc. 20, 309–325 (2021). 10.1038/s41573-020-00135-8 33536635

[2] Cooper B. M., Iegre J., O’Donovan D. H., Halvarsson M. Ö. & Spring D. R. Peptides as a platform for targeted therapeutics for cancer: peptide–drug conjugates (PDCs). Chem. Soc. Rev. 50, 1480–1494 (2021). 33346298 10.1039/d0cs00556h

[3] Wang L. Therapeutic peptides: current applications and future directions. Signal Transduct. 7, 48 (2022). 10.1038/s41392-022-00904-4 PMC8844085 35165272

[4] Fetse J., Kandel S., Mamani U.-F. & Cheng K. Recent advances in the development of therapeutic peptides. Trends Pharmacol. Sci. 44, 425–441 (2023). 37246037 10.1016/j.tips.2023.04.003 PMC10330351

[5] Barman P. Strategic approaches to improvise peptide drugs as next generation therapeutics. Int. J. Pept. Res. Ther. 29, 61 (2023). 37251528 10.1007/s10989-023-10524-3 PMC10206374

[6] Sharma K., Sharma K. K., Sharma A. & Jain R. Peptide-based drug discovery: Current status and recent advances. Drug Discov. Today 28, 103464 (2023). 36481586 10.1016/j.drudis.2022.103464

[7] Hickey J. L., Sindhikara D., Zultanski S. L. & Schultz D. M. Beyond 20 in the 21st century: prospects and challenges of non-canonical amino acids in peptide drug discovery. ACS Med. Chem. Lett. 14, 557–565 (2023). 37197469 10.1021/acsmedchemlett.3c00037 PMC10184154

[8] Zhang H. & Chen S. Cyclic peptide drugs approved in the last two decades (2001–2021). RSC Chem. Biol. 3, 18–31 (2022). 35128405 10.1039/d1cb00154j PMC8729179

[9] Ji X., Nielsen A. L. & Heinis C. Cyclic peptides for drug development. Angew. Chem. Int. Ed. 202308251, e202308251 (2023). 10.1002/anie.202308251 37870189

[10] Lamers C. Overcoming the shortcomings of peptide-based therapeutics. Future Drug Discov. 4, FDD75 (2022).

[11] Openy J. Backbone alterations in cyclic peptides influence both membrane permeability and biological activity. J. Med. Chem. 68, 24108–24126 (2025). 41196074 10.1021/acs.jmedchem.5c01901 PMC12670408

[12] Castro T. G., Melle-Franco M., Sousa C. E., Cavaco-Paulo A. & Marcos J. C. Non-canonical amino acids as building blocks for peptidomimetics: Structure, function, and applications. Biomolecules 13, 981 (2023). 37371561 10.3390/biom13060981 PMC10296201

[13] Terasaka N., Iwane Y., Geiermann A.-S., Goto Y. & Suga H. Recent developments of engineered translational machineries for the incorporation of non-canonical amino acids into polypeptides. International Journal of Molecular Sciences 16, 6513–6531 (2015). 25803109 10.3390/ijms16036513 PMC4394545

[14] Guo Z. & Diao T. Late-stage serine modification enables noncanonical peptide synthesis. Journal of the American Chemical Society 147, 33127–33135 (2025). 40853838 10.1021/jacs.5c11065 PMC12426918

[15] Du Z., Caragea D., Guo X. & Li Y. PepBERT: Lightweight language models for bioactive peptide representation. bioRxiv 10.1101/2025.04.08.647838 (2025).

[16] Wang L. PepDoRA: A unified peptide language model via weight-decomposed low-rank adaptation. arXiv 2410.20667 (2024).

[17] Fernández-Díaz R., Ochoa R., Hoang T. L., Lopez V. & Shields D. How to build machine learning models able to extrapolate from standard to modified peptides. J. Cheminform. (2025). 10.1186/s13321-025-01115-z PMC12751563 41310881

[18] Consortium T. U. UniProt: the universal protein knowledgebase in 2025. Nucleic Acids Res. 53, D609–D617 (2025). 39552041 10.1093/nar/gkae1010 PMC11701636

[19] Rives A. Biological structure and function emerge from scaling unsupervised learning to 250 million protein sequences. Proc. Natl. Acad. Sci. 118, e2016239118 (2021). 33876751 10.1073/pnas.2016239118 PMC8053943

[20] Lin Z. Evolutionary-scale prediction of atomic-level protein structure with a language model. Science 379, 1123–1130 (2023). 36927031 10.1126/science.ade2574

[21] Hayes T. Simulating 500 million years of evolution with a language model. Science 387, 850–858 (2025). 39818825 10.1126/science.ads0018

[22] Elnaggar A. ProtTrans: towards cracking the language of life’s code through self-supervised learning. IEEE Trans. Pattern Anal. Mach. Intell. 44, 7112–7127 (2021). 10.1109/TPAMI.2021.3095381 34232869

[23] Alanazi W., Meng D. & Pollastri G. Porter 6: protein secondary structure prediction by leveraging pre-trained language models (PLMs). Int. J. Mol. Sci. 26, 130 (2024). 39795988 10.3390/ijms26010130 PMC11719765

[24] Zhang Z. Protein language models learn evolutionary statistics of interacting sequence motifs. Proc. Natl. Acad. Sci. 121, e2406285121 (2024). 39467119 10.1073/pnas.2406285121 PMC11551344

[25] Heinzinger M. Contrastive learning on protein embeddings enlightens midnight zone. NAR Genom. Bioinform. 4, lqac043 (2022). 35702380 10.1093/nargab/lqac043 PMC9188115

[26] Sun Y. & Shen Y. Structure-informed protein language models are robust predictors for variant effects. Hum. Genet. 144, 209–225 (2025). 39117802 10.1007/s00439-024-02695-w PMC12068927

[27] Gurev S., Youssef N., Jain N. & Marks D. Variant effect prediction with reliability estimation across priority viruses. bioRxiv 10.1101/2025.08.04.668549 (2025).

[28] Liu D. PLM-interact: extending protein language models to predict protein-protein interactions. Nat. Commun. 16, 9012 (2025). 41145424 10.1038/s41467-025-64512-w PMC12559430

[29] Lu A. X., Zhang H., Ghassemi M. & Moses A. Self-supervised contrastive learning of protein representations by mutual information maximization. BioRxiv 10.1101/2020.09.04.283929 (2020).

[30] Weininger D. SMILES, a chemical language and information system. J. Chem. Inf. Comput. Sci. 28, 31–36 (1988).

[31] Krenn M. SELFIES and the future of molecular string representations. Patterns 3, 100588 (2022). 36277819 10.1016/j.patter.2022.100588 PMC9583042

[32] Wang S., Guo Y., Wang Y., Sun H. & Huang J. SMILES-BERT: Large-scale unsupervised pre-training for molecular property prediction. Proc. ACM Conf. Bioinform. Comput. Biol. 429–436 (2019).

[33] Chithrananda S., Grand G. & Ramsundar B. ChemBERTa: large-scale self-supervised pretraining for molecular property prediction. arXiv 2010.09885 (2020).

[34] Ross J. Large-scale chemical language representations capture molecular structure and properties. Nat. Mach. Intell. 4, 1256–1264 (2022).

[35] Park J.-H., Park H., Kim Y., Lim W. & Lee S. Moleco: Molecular contrastive learning with chemical language models for molecular property prediction. Proceedings of the 2024 Conference on Empirical Methods in Natural Language Processing: Industry Track 408–420 (2024).

[36] Park J.-H., Kim Y., Lee M., Park H. & Lee S. Moltres: Improving chemical language representation learning for molecular property prediction. arXiv preprint arXiv:2408.01426 (2024).

[37] Ahmad W., Simon E., Chithrananda S., Grand G. & Ramsundar B. ChemBERTa-2: Towards chemical foundation models. arXiv 2209.01712 (2022).

[38] Lv L. ProLLaMA: A protein large language model for multi-task protein language processing. IEEE Trans. Artif. Intell. (2025).

[39] Praski M., Adamczyk J. & Czech W. Benchmarking pretrained molecular embedding models for molecular representation learning. arXiv 2508.06199 (2025).

[40] Adamczyk J., Ludynia P. & Czech W. Molecular fingerprints are strong models for peptide function prediction. arXiv 2501.17901 (2025). 10.1093/bioinformatics/btag179 PMC13143419 41981726

[41] Devlin J., Chang M.-W., Lee K. & Toutanova K. BERT: Pre-training of deep bidirectional transformers for language understanding. Proc. NAACL 4171–4186 (2019).

[42] Feller A. L. & Wilke C. O. Peptide-aware chemical language model successfully predicts membrane diffusion of cyclic peptides. J. Chem. Inf. Model. 65, 571–579 (2025). 39772542 10.1021/acs.jcim.4c01441 PMC11971985

[43] Borchani H., Varando G., Bielza C. & Larranaga P. A survey on multi-output regression. WIREs Data Min. Knowl. Discov. 5, 216–233 (2015).

[44] Landrum G. RDKit: Open-source cheminformatics (2013). URL http://www.rdkit.org. Online.

[45] Su J. Enhanced transformer with rotary position embedding., 2021. DOI: 10.1016/j.neucom (2023).

[46] Elfwing S., Uchibe E. & Doya K. Sigmoid-weighted linear units for neural network function approximation in reinforcement learning. Neural Netw. 107, 3–11 (2018). 29395652 10.1016/j.neunet.2017.12.012

[47] Xiong R. On layer normalization in the transformer architecture. Proc. Int. Conf. Mach. Learn. (ICML) 10524–10533 (2020).

[48] Joshi M. SpanBERT: Improving pre-training by representing and predicting spans. Trans. Assoc. Comput. Linguist. 8, 64–77 (2020).

[49] Sud M. LMSD: LIPID MAPS structure database. Nucleic Acids Res. 35, D527–D532 (2007). 17098933 10.1093/nar/gkl838 PMC1669719

[50] Kim S. PubChem 2025 update. Nucleic Acids Res. 53, D1516–D1525 (2025). 39558165 10.1093/nar/gkae1059 PMC11701573

[51] Morgan H. L. The generation of a unique machine description for chemical structures-a technique developed at Chemical Abstracts Service. J. Chem. Doc. 5, 107–113 (1965).

[52] Ramsundar B. Deep Learning for the Life Sciences (O’Reilly, 2019).

[53] Li J. CycPeptMPDB: a comprehensive database of membrane permeability of cyclic peptides. J. Chem. Inf. Model. 63, 2240–2250 (2023). 36930969 10.1021/acs.jcim.2c01573 PMC10091415

[54] Scodeller P. & Asciutto E. K. Targeting tumors using peptides. Molecules 25, 808 (2020). 32069856 10.3390/molecules25040808 PMC7070747

[55] Shoombuatong W., Schaduangrat N., Pratiwi R. & Nantasenamat C. THPep: A machine learning-based approach for predicting tumor homing peptides. Comput. Biol. Chem. 80, 441–451 (2019). 31151025 10.1016/j.compbiolchem.2019.05.008

[56] Kumar V. Prediction of cell-penetrating potential of modified peptides containing natural and chemically modified residues. Front. Microbiol. 9, 725 (2018). 29706944 10.3389/fmicb.2018.00725 PMC5906597

[57] He Y., Song X., Wan H. & Zhao X. AmpHGT: expanding prediction of antimicrobial activity in peptides containing non-canonical amino acids using multi-view constrained heterogeneous graph transformer. BMC Biol. 23, 184 (2025). 40598389 10.1186/s12915-025-02253-4 PMC12217533

[58] Xue C., Lin T. Y., Chang D. & Guo Z. Thioflavin T as an amyloid dye: fibril quantification, optimal concentration and effect on aggregation. R. Soc. Open Sci. 4, 160696 (2017). 28280572 10.1098/rsos.160696 PMC5319338

[59] Quartararo A. J. Ultra-large chemical libraries for the discovery of high-affinity peptide binders. Nat. Commun. 11, 3183 (2020). 32576815 10.1038/s41467-020-16920-3 PMC7311396

[60] Niquille D. L. Nonribosomal biosynthesis of backbone-modified peptides. Nat. Chem. 10, 282–287 (2018). 29461527 10.1038/nchem.2891

[61] Shin J.-E. Protein design and variant prediction using autoregressive generative models. Nat. Commun. 12, 2403 (2021). 33893299 10.1038/s41467-021-22732-w PMC8065141

[62] Feller A. L. & Wilke C. O. p2smi: A toolkit enabling smiles generation and property analysis for noncanonical and cyclized peptides. Journal of Open Source Software 10, 8319 (2025).

[63] Li X. & Fourches D. SMILES pair encoding: a data-driven substructure tokenization algorithm for deep learning. J. Chem. Inf. Model. 61, 1560–1569 (2021). 33715361 10.1021/acs.jcim.0c01127

[64] Li Y. SmProt: a reliable repository with comprehensive annotation of small proteins identified from ribosome profiling. Genomics Proteomics Bioinformatics 19, 602–610 (2021). 34536568 10.1016/j.gpb.2021.09.002 PMC9039559

[65] Arús-Pous J. Randomized SMILES strings improve the quality of molecular generative models. J. Cheminform. 11, 71 (2019). 33430971 10.1186/s13321-019-0393-0 PMC6873550

[66] Pedregosa F. Scikit-learn: Machine learning in Python. J. Mach. Learn. Res. 12, 2825–2830 (2011)..

