# Supplementary material for: Scaling SMILES-based chemical language models for therapeutic peptide engineering
Source: bioRxiv. 2026 Jun 23:2026.01.06.697994. Originally published 2026 Jan 7. Preprint. [Version 5] doi: 10.64898/2026.01.06.697994 (PMC12803269; doi:10.64898/2026.01.06.697994)
Supplement: Supplement 1 [file media-1.pdf]

Supplementary Information:
Scaling SMILES-based chemical language models for therapeutic peptide engineering

Aaron L. Feller[1,2], **Maxim Secor**[2], **Sebastian Swanson**[2], **Claus O. Wilke**[1], and **Kristine Diebler**[2]

[1]Integrative Biology, The University of Texas at Austin, Austin, TX 78712, USA
[2]Molecular AI, Novo Nordisk, Lexington, MA 02421, USA

Contents

Supplementary Tables **S2**

S1: *RDKit Descriptors by Type* S2
S2: *Kmer Filtering Rules* S3
S3: *Sample Token Vocabulary* S3
S4: *Pretraining Parameters* S4
S5: *Downstream Workflow Settings* S5
S6: *Finetuning Regularization Mappings* S6
S7: *Permeability Benchmark Statistics* S6
S8: *Tumor-Homing Benchmark Metrics* S7
S9: *Cell-Penetration Benchmark Metrics* S7
S10: *Antimicrobial Benchmark Metrics* S7
S11: *PepMSND Blood Stability Statistics* S7

Supplementary Figures **S8**

S1: *t-SNE Embeddings Visualizations* S8
S2: *Layer Probing Profiles* S9

Supplementary Tables

Table S1: RDKit Descriptors Organized by Type

Descriptor Type	Descriptors
**Topological Indices**	Chi0, Chi0n, Chi0v, Chi1, Chi1n, Chi1v, Chi2n, Chi2v
**Morgan Fingerprints**	FpDensityMorgan1, FpDensityMorgan2, FpDensityMorgan3
**Kappa Indices**	Kappa1, Kappa2, Kappa3
**Molecular Properties**	ExactMolWt, MaxAbsPartialCharge, MaxPartialCharge, MinAbsPartialCharge, MinPartialCharge, MolLogP, MolMR, MolWt
**Structural Counts**	RingCount, HeavyAtomCount, HeavyAtomMolWt, FractionCSP3
**SA Descriptor**	HallKierAlpha, LabuteASA, TPSA
**Atom Count Descriptors**	NHOHCount, NOCount, NumHAcceptors, NumHDonors, NumHeteroatoms
**Ring Counts**	NumAliphaticCarbocycles, NumAliphaticHeterocycles, NumAliphaticRings, NumAromaticCarbocycles, NumAromaticHeterocycles, NumAromaticRings
**Electronic Descriptors**	NumRadicalElectrons, NumValenceElectrons
**Rotatable Bonds**	NumRotatableBonds
**Saturated Structure Counts**	NumSaturatedCarbocycles, NumSaturatedHeterocycles, NumSaturatedRings
**PEOE Descriptors**	PEOE_VSA1, PEOE_VSA2, PEOE_VSA3, PEOE_VSA4, PEOE_VSA5, PEOE_VSA6, PEOE_VSA7, PEOE_VSA8, PEOE_VSA9, PEOE_VSA10, PEOE_VSA11, PEOE_VSA12, PEOE_VSA13, PEOE_VSA14
**SMR Descriptors**	SMR_VSA1, SMR_VSA2, SMR_VSA3, SMR_VSA4, SMR_VSA5, SMR_VSA6, SMR_VSA7, SMR_VSA8, SMR_VSA9, SMR_VSA10
**SlogP Descriptors**	SlogP_VSA1, SlogP_VSA2, SlogP_VSA3, SlogP_VSA4, SlogP_VSA5, SlogP_VSA6, SlogP_VSA7, SlogP_VSA8, SlogP_VSA9, SlogP_VSA10, SlogP_VSA11, SlogP_VSA12
**Functional Groups**	fr_amide, fr_NH0, fr_NH1, fr_NH2, fr_COO, fr_priamide, fr_guanido, fr_imidazole, fr_phenol, fr_Al_OH, fr_C_O, fr_ether, fr_alkyl_halide, fr_unbrch_alkane, fr_aryl_methyl, fr_benzene, fr_ester, fr_ketone, fr_methoxy, fr_sulfide, fr_sulfonamd

Table S2: Filtering rules applied during construction of the kmer vocabulary.

#	**Remove tokens:**
1	with numbers
2	that start with `')'`
3	that end with `'('`
4	that contain `')*'` w/o a leading `'*('`
5	with more than 4 atom characters
6	with fewer than 1,000 occurrences

Table S3: Sample tokens extracted from the initialized kmer tokenizer.

**Tokens**
=NC, #Cc, c(CO), n(C)c, n(C), CN=C, (CCO), CCO), C(C)N, C#C, C(CC), CS(=O), NC(C), CSc, NN, CC(O), CNc, CCn

Table S4: Pretraining model configurations and hardware optimization mappings.

Setting	Value
Architecture	BERT-style encoder (Small/Base/Large)
Parameter scales	32M, 114M, 337M
Small architecture	14 blocks, embed 512, 8 heads, FFN 768
Base architecture	24 blocks, embed 768, 12 heads, FFN 1024
Large architecture	32 blocks, embed 1024, 16 heads, FFN 2048
Tokenizer	kmer SMILES (vocab: 405)
Max sequence length	2048
Batch size	512 seqs (global)
Optimizer	AdamW
AdamW ($\beta_1, \beta_2, \epsilon$)	$(0.9, 0.98, 1\text{e}{-8})$
Weight decay	0.01
Learning rate (peak)	3e−4
LR schedule	Cosine decay with linear warmup
Warmup steps	5,000
Training steps	100k
Dropout (attn/ffn)	0.1 / 0.1
Masking rate (MLM)	25%
Span masking	$\mathcal{N}(\mu = 3.5, \sigma = 1)$ spans
MTR heads	2-layer MLP, SiLU, mean-pooled embedding
Regression targets	99 RDKit descriptors (normalized)
Loss weights	$\lambda_{\text{MLM}} = 0.6,\ \lambda_{\text{MTR}} = 0.4$
Precision	bfloat16
Gradient clipping	0.1
Gradient accumulation	1 / 2 / 4 (small/base/large)
Data aug.	RDKit randomized SMILES

Table S5: Released downstream training settings organized by validation workflow.

Workflow	Released setting
CycPeptMPDB regression	Mean-pooled transformer embeddings with a small regression head; MSE loss; AdamW; batch size 16; max 10 epochs; validation every 0.2 epoch; early stopping on validation loss; outer held-out folds with inner-fold ensembling.
Classification benchmarks	Rank-16 LoRA adapters on the attention projection layers with a SiLU projection head; AdamW; default batch size 16; linear warmup scheduler; max 10 epochs; early stopping patience 5; fixed splits when available and five-fold CV otherwise.
ChemBERTa baseline	Batch size 8; learning rate 2×10^{-5}; max 8 epochs; early stopping patience 2.
PepMSND	Mean-pooled transformer embedding fused with one-hot Species/Environment covariates; BCEWithLogitsLoss; AdamW with learning rate 1×10^{-5}; batch size 32; max 10 epochs; early stopping patience 3.
Reported summaries	Classification metrics: MCC, AUROC, F1. Regression metrics: R^2, RMSE, MAE. Publication figures aggregate repeated runs across three random seeds unless otherwise noted.

Table S6: Finetuning optimization criteria and validation dimensions.

Setting	Value (Small, Base, Large)
Task heads	Regression
Loss	MSE
Batch size	16
Learning rate	3e−4
Optimizer	AdamW
Weight decay	0.01
LR schedule	No decay; no warmup
Dropout (head)	0.1
Max epochs (per fold)	10
Evaluation	20% epoch (early stopping patience 3)
CV scheme	Outer holdout + inner K-fold
Ensembling	Mean of checkpoints across inner folds
Class imbalance	Equal sampling across bins
Input length	No truncations required
Replicates	3 (report mean ± std)

Table S7: CycPeptMPDB test-set regression metrics from the released triplicate permeability runs, reported as mean ± SD.

Size / Control	Released setting	R^2	RMSE	MAE
Control	Random initialization	-0.079 ± 0.048	0.805 ± 0.018	0.622 ± 0.010
**Small**	MLM	0.237 ± 0.140	0.675 ± 0.061	0.516 ± 0.052
	Hybrid	0.232 ± 0.095	0.678 ± 0.042	0.527 ± 0.037
	**MTR**	$\mathbf{0.378 \pm 0.048}$	$\mathbf{0.611 \pm 0.024}$	$\mathbf{0.467 \pm 0.025}$
**Base**	MLM	0.489 ± 0.086	0.553 ± 0.048	0.420 ± 0.037
	Hybrid	0.495 ± 0.042	0.550 ± 0.023	0.419 ± 0.017
	**MTR**	$\mathbf{0.502 \pm 0.034}$	$\mathbf{0.547 \pm 0.019}$	$\mathbf{0.412 \pm 0.017}$
**Large**	MLM	0.582 ± 0.041	0.500 ± 0.025	0.374 ± 0.018
	**Hybrid**	$\mathbf{0.613 \pm 0.044}$	$\mathbf{0.482 \pm 0.028}$	$\mathbf{0.361 \pm 0.022}$
	MTR	0.582 ± 0.018	0.501 ± 0.011	0.374 ± 0.010

Table S8: THPep tumor-homing test-set metrics across three random seeds for the released benchmark models, reported as mean ± SD.

Model	MCC	AUROC	F1
RDKit99	0.673 ± 0.092	0.932 ± 0.029	0.766 ± 0.066
MorganFP	0.597 ± 0.040	0.903 ± 0.009	0.707 ± 0.027
ChemBERTa-2	0.618 ± 0.118	0.921 ± 0.013	0.719 ± 0.105
CheMeleon	0.600 ± 0.062	0.888 ± 0.049	0.703 ± 0.051
PeptideCLM-2 (MLM)	$\mathbf{0.756 \pm 0.019}$	$\mathbf{0.949 \pm 0.006}$	$\mathbf{0.826 \pm 0.012}$
PeptideCLM-2 (Hybrid)	0.747 ± 0.036	0.940 ± 0.019	0.818 ± 0.022
PeptideCLM-2 (MTR)	0.698 ± 0.036	0.924 ± 0.016	0.784 ± 0.029

Table S9: CellPPD-Mod cell-penetrating test-set metrics across three random seeds for the released benchmark models, reported as mean ± SD.

Model	MCC	AUROC	F1
RDKit99	0.829 ± 0.010	0.968 ± 0.001	0.915 ± 0.005
MorganFP	0.891 ± 0.003	$\mathbf{0.992 \pm 0.000}$	0.942 ± 0.002
ChemBERTa-2	0.768 ± 0.008	0.943 ± 0.004	0.876 ± 0.004
CheMeleon	$\mathbf{0.892 \pm 0.016}$	0.983 ± 0.000	$\mathbf{0.945 \pm 0.007}$
PeptideCLM-2 (MLM)	0.875 ± 0.004	0.981 ± 0.002	0.935 ± 0.003
PeptideCLM-2 (Hybrid)	0.860 ± 0.003	0.976 ± 0.001	0.926 ± 0.003
PeptideCLM-2 (MTR)	0.867 ± 0.004	0.975 ± 0.001	0.929 ± 0.002

Table S10: AMP-HGT antimicrobial test-set metrics across three random seeds for the released benchmark models, reported as mean ± SD.

Model	MCC	AUROC	F1
RDKit99	0.835 ± 0.007	0.967 ± 0.002	0.923 ± 0.003
MorganFP	0.836 ± 0.005	$\mathbf{0.986 \pm 0.001}$	0.923 ± 0.002
ChemBERTa-2	0.814 ± 0.010	0.957 ± 0.007	0.914 ± 0.004
CheMeleon	0.818 ± 0.009	0.965 ± 0.002	0.916 ± 0.004
PeptideCLM-2 (MLM)	$\mathbf{0.884 \pm 0.013}$	0.980 ± 0.004	$\mathbf{0.946 \pm 0.006}$
PeptideCLM-2 (Hybrid)	0.850 ± 0.014	0.972 ± 0.003	0.930 ± 0.007
PeptideCLM-2 (MTR)	0.853 ± 0.039	0.972 ± 0.011	0.931 ± 0.018

Table S11: PepMSND test-set metrics from the released triplicate prediction exports, reported as mean ± SD across runs.

Released model	MCC	AUROC	F1
MLM-MTR embedding + linear head	0.419 ± 0.026	0.791 ± 0.007	0.598 ± 0.039
MLM-MTR embedding + KAN head	$\mathbf{0.640 \pm 0.016}$	$\mathbf{0.879 \pm 0.002}$	$\mathbf{0.788 \pm 0.008}$

Supplementary Figures

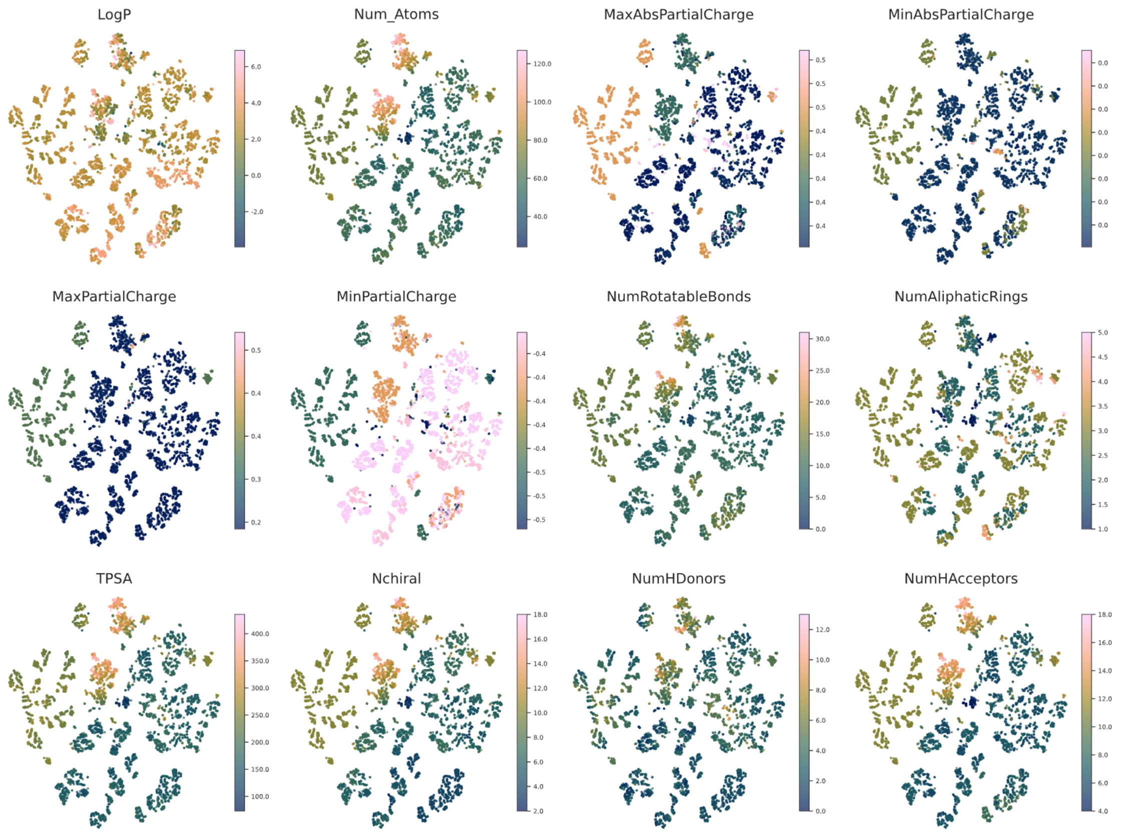

Figure S1: **Latent space visualization of key molecular descriptors.** 2D t-SNE plots of PeptideMLM embeddings (large model) displayed with colorbars of with calculated physicochemical properties. Clustering demonstrates the correlation between the learned embedding space and specific chemical features including physical and chemical properties.

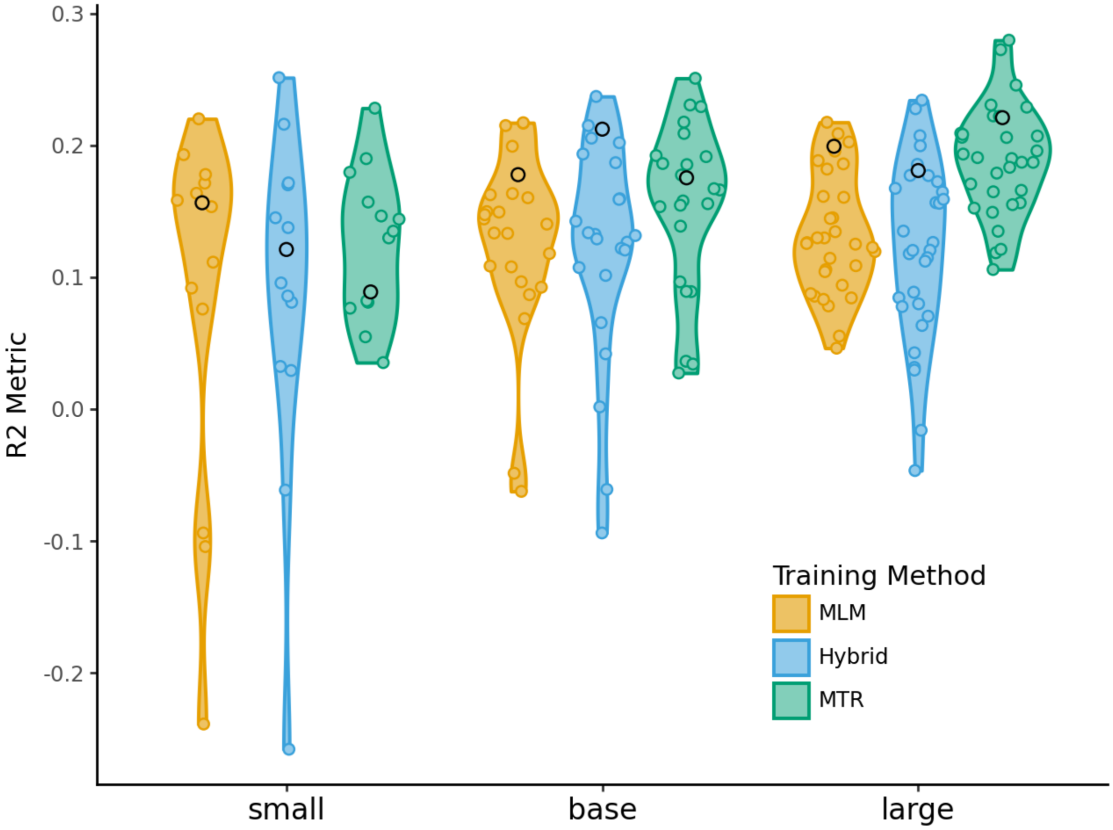

Figure S2: **Linear probing of embeddings for PAMPA permeability.** Violin plots displaying the distribution of R^2 scores obtained via LassoCV regression on the CycPeptMPDB dataset. Models were trained using mean-pooled embeddings extracted from pre-trained encoders, stratified by architecture size (Small, Base, Large) and training objective (MLM, Hybrid, MTR). The spread within each violin represents the variance in predictive power across embeddings extracted from different model layers. The final layer is indicated with a black dot.
