# Supplementary material for: Scaling SMILES-based chemical language models for therapeutic peptide engineering
Source: bioRxiv. 2026 Apr 17:2026.01.06.697994. Originally published 2026 Jan 7. Preprint. [Version 3] doi: 10.64898/2026.01.06.697994 (PMC12803269; doi:10.64898/2026.01.06.697994)
Supplement: Supplementary file 1 [file media-1.pdf]

Supplemental information for Scaling SMILES-Based Chemical Language Models for Therapeutic Peptide Engineering

Aaron L. Feller[1,2*], Maxim Secor[1], Sebastian Swanson[1], Claus O. Wilke[2], Kristine Diebler[1]

[1]Molecular AI, Novo Nordisk, 33 Hayden Ave, Lexington, 02421, MA, USA.
[2]Integrative Biology, The University of Texas at Austin, 2500 Speedway, Austin, 78712, TX, USA.

*Corresponding author(s). E-mail(s): aaron.feller@utexas.edu;
Contributing authors: olsw@novonordisk.com; sbxw@novonordisk.com; wilke@austin.utexas.edu; ktdb@novonordisk.com;

Contents

S1 Supplementary Tables **2**
S1.1 RDKit descriptors for pretraining 2
S1.2 Kmer filtering 3
S1.3 Example tokens 3
S1.4 Pretraining hyperparameters 4
S1.5 Finetuning hyperparameters 5
S1.6 Membrane permeability prediction 6
S1.7 Classification predictions 6

S2 Supplementary Figures **7**
S2.1 Latent space visualization 7
S2.2 Linear layer probing 8

S1 Supplementary Tables

S1.1 RDKit descriptors for pretraining

Table S1: RDKit Descriptors Organized by Type

Descriptor Type	Descriptors
1. **Topological Indices**	Chi0, Chi0n, Chi0v, Chi1, Chi1n, Chi1v, Chi2n, Chi2v
2. **Morgan Fingerprints**	FpDensityMorgan1, FpDensityMorgan2, FpDensityMorgan3
3. **Kappa Indices**	Kappa1, Kappa2, Kappa3
4. **Molecular Properties**	ExactMolWt, MaxAbsPartialCharge, MaxPartialCharge, MinAbsPartialCharge, MinPartialCharge, MolLogP, MolMR, MolWt
5. **Structural Counts**	RingCount, HeavyAtomCount, HeavyAtomMolWt, FractionCSP3
6. **SA Descriptor**	HallKierAlpha, LabuteASA, TPSA
7. **Atom Count Descriptors**	NHOHCount, NOCount, NumHAcceptors, NumHDonors, NumHeteroatoms
8. **Ring Counts**	NumAliphaticCarbocycles, NumAliphaticHeterocycles, NumAliphaticRings, NumAromaticCarbocycles, NumAromaticHeterocycles, NumAromaticRings
9. **Electronic Descriptors**	NumRadicalElectrons, NumValenceElectrons
10. **Rotatable Bonds**	NumRotatableBonds
11. **Saturated Structure Counts**	NumSaturatedCarbocycles, NumSaturatedHeterocycles, NumSaturatedRings
12. **PEOE Descriptors**	PEOE_VSA1, PEOE_VSA2, PEOE_VSA3, PEOE_VSA4, PEOE_VSA5, PEOE_VSA6, PEOE_VSA7, PEOE_VSA8, PEOE_VSA9, PEOE_VSA10, PEOE_VSA11, PEOE_VSA12, PEOE_VSA13, PEOE_VSA14
13. **SMR Descriptors**	SMR_VSA1, SMR_VSA2, SMR_VSA3, SMR_VSA4, SMR_VSA5, SMR_VSA6, SMR_VSA7, SMR_VSA8, SMR_VSA9, SMR_VSA10
14. **SlogP Descriptors**	SlogP_VSA1, SlogP_VSA2, SlogP_VSA3, SlogP_VSA4, SlogP_VSA5, SlogP_VSA6, SlogP_VSA7, SlogP_VSA8, SlogP_VSA9, SlogP_VSA10, SlogP_VSA11, SlogP_VSA12
15. **Functional Groups**	fr_amide, fr_NH0, fr_NH1, fr_NH2, fr_COO, fr_priamide, fr_guanido, fr_imidazole, fr_phenol, fr_Al_OH, fr_C_O, fr_ether, fr_alkyl_halide, fr_unbrch_alkane, fr_aryl_methyl, fr_benzene, fr_ester, fr_ketone, fr_methoxy, fr_sulfide, fr_sulfonamd

S1.2 Kmer filtering

Table S2: Filtering rules applied during construction of the kmer vocabulary.

#	**Remove tokens:**
1	with numbers
2	that start with `')'`
3	that end with `'('`
4	that contain `')*'` w/o a leading `'*('`
5	with more than 4 atom characters
6	with fewer than 1,000 occurrences

S1.3 Example tokens

Table S3: Sample tokens from kmer tokenizer.

**Tokens**
=NC, #Cc, c(CO), n(C)c, n(C), CN=C, (CCO), CCO), C(C)N, C#C, C(CC), CS(=O), NC(C), CSc, NN, CC(O), CNc, CCn

S1.4 Pretraining hyperparameters

Table S4: Pretraining hyperparameters.

Setting	Value
Architecture	BERT-style encoder (Small/Base/Large)
Parameters	32M, 114M, 337M
Tokenizer	kmer SMILES (vocab: 405)
Max sequence length	2048
Batch size	512 seqs (global)
Optimizer	AdamW
AdamW $(\beta_1, \beta_2, \epsilon)$	$(0.9, 0.999, 1\text{e}{-8})$
Weight decay	0.01
Learning rate (peak)	3e−4
LR schedule	Cosine decay with linear warmup
Warmup steps	5,000
Training steps	100k
Dropout (attn/ffn)	0.1 / 0.1
Masking rate (MLM)	25%
Span masking	$\mathcal{N}(\mu{=}3.5, \sigma{=}1)$ spans
MTR heads	2-layer MLP, SiLU, mean-pooled embedding
Regression targets	99 RDKit descriptors (normalized)
Loss weights	$\lambda_{\text{MLM}} = 0.6,\ \lambda_{\text{MTR}} = 0.4$
Precision	bfloat16
Gradient clipping	0.1
Gradient accumulation	1–8 (as needed for memory)
Data aug.	RDKit randomized SMILES

S1.5 Finetuning hyperparameters

Table S5: Finetuning hyperparameters (nested CV + ensembling).

Setting	Value (Small, Base, Large)
Task heads	Regression
Loss	MSE
Batch size	16
Learning rate	3e−4
Optimizer	AdamW
Weight decay	0.01
LR schedule	No decay; no warmup
Dropout (head)	0.1
Max epochs (per fold)	10
Evaluation	20% epoch (early stopping patience 3)
CV scheme	Outer holdout + inner K-fold
Ensembling	Mean of checkpoints across inner folds
Class imbalance	Equal sampling across bins
Input length	No truncations required
Replicates	3 (report mean $\pm$ std)

S1.6 Membrane permeability prediction

Table S6: Ablation studies of PeptideMTR models on cyclic peptide permeability prediction, ordered by model size and performance.

Size	Tokenizer	Masking	Training	R^2	RMSE	AUROC	AUPRC
**Small**	Kmer	*	*	−0.18	0.84	0.63	0.59
	Kmer	Span	MLM	0.13	0.72	0.68	0.69
	Kmer	Span	MLM-MTR	0.23	0.68	0.70	0.74
	Kmer	Random	MLM-MTR	0.24	0.68	0.70	0.74
	Atom	Random	MLM-MTR	0.25	0.67	0.71	0.74
	Kmer	None	**MTR**	**0.38**	**0.61**	**0.76**	**0.73**
**Base**	Kmer	*	*	−0.24	0.86	0.62	0.55
	Kmer	Random	MLM-MTR	0.39	0.59	0.79	0.77
	Kmer	Span	MLM	0.47	0.56	0.80	0.77
	Kmer	None	MTR	0.50	0.55	0.80	0.78
	Kmer	Span	**MLM-MTR**	**0.52**	**0.54**	**0.81**	**0.78**
**Large**	Kmer	*	*	−0.17	0.84	0.56	0.50
	Kmer	Random	MLM-MTR	0.57	0.51	0.83	0.81
	Kmer	Span	**MLM**	**0.58**	**0.50**	**0.83**	**0.81**
	Kmer	None	**MTR**	**0.58**	**0.50**	**0.83**	**0.81**
	Kmer	Span	**MLM-MTR**	**0.58**	**0.50**	**0.83**	**0.81**

* model randomly initialized prior to finetuning

S1.7 Classification predictions

Table S7: Performance of PeptideMTR compared to published baseline for therapeutic peptide property prediction.

Dataset	Target	Prior	MFP	MLM	MLM-MTR	MTR
THPep	Tumor Homing (MCC)	0.71	0.478	**0.732**	0.698	0.680
CellPPD-Mod	Cell Penetrating (MCC)	0.85	0.772	0.868	**0.875**	0.856
AMP-HGT	Antimicrobial (MCC)	0.797	**0.813**	**0.813**	0.771	0.735

S2 Supplementary Figures

S2.1 Latent space visualization

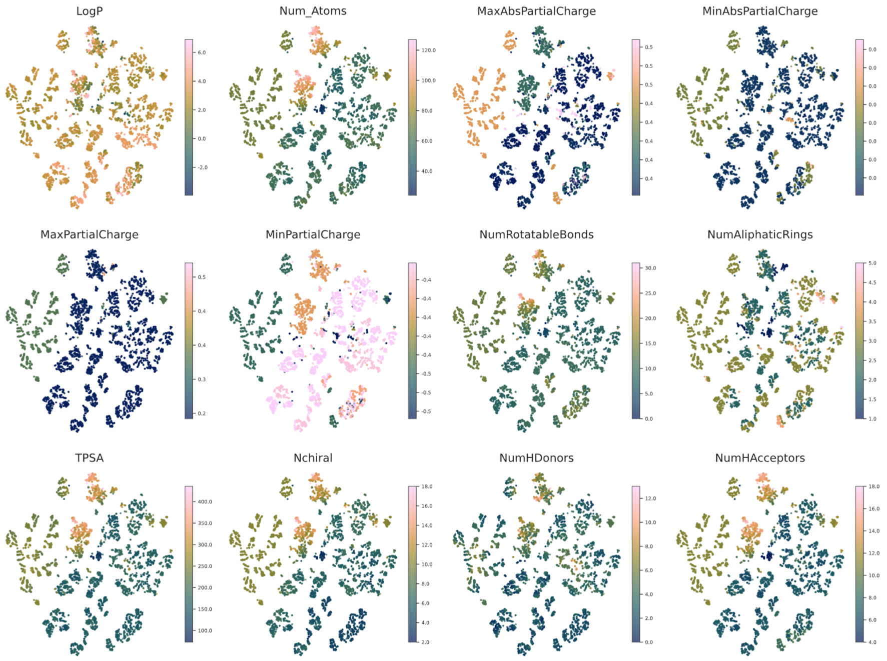

Fig. S1: **Latent space visualization of key molecular descriptors.** 2D t-SNE plots of PeptideMLM embeddings (large model) displayed with colorbars of with calculated physicochemical properties. Clustering demonstrates the correlation between the learned embedding space and specific chemical features including physical and chemical properties.

S2.2 Linear layer probing

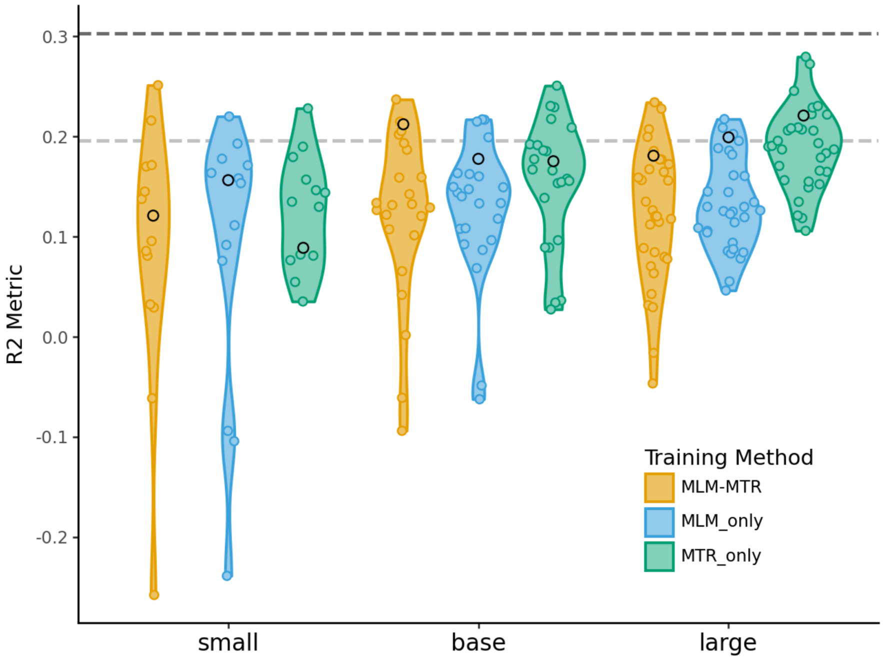

Fig. S2: **Linear probing of embeddings for PAMPA permeability.** Violin plots displaying the distribution of R^2 scores obtained via LassoCV regression on the CycPeptMPDB dataset. Models were trained using mean-pooled embeddings extracted from pre-trained encoders, stratified by architecture size (Small, Base, Large) and training objective (MLM-MTR, MLM-only, MTR-only). The spread within each violin represents the variance in predictive power across embeddings extracted from different model layers. Last layer is indicated with a black dot
